# The Peptide Antibiotic Corramycin Adopts a β-Hairpin-like Structure and Is Inactivated by the Kinase ComG

**DOI:** 10.1021/jacs.3c13208

**Published:** 2024-03-21

**Authors:** Sebastian Adam, Franziska Fries, Alexander von Tesmar, Sari Rasheed, Selina Deckarm, Carla F. Sousa, Roman Reberšek, Timo Risch, Stefano Mancini, Jennifer Herrmann, Jesko Koehnke, Olga V. Kalinina, Rolf Müller

**Affiliations:** † Helmholtz Institute for Pharmaceutical Research Saarland (HIPS), Helmholtz Centre for Infection Research (HZI), Saarland University, Campus E8 1, 66123 Saarbrücken, Germany; ○ Department of Pharmacy, Saarland University, 66123 Saarbrücken, Germany; § German Center for Infection Research (DZIF), Partner Site Hannover-Braunschweig, 38124 Braunschweig, Germany; ∥ Institute of Medical Microbiology, University of Zürich, 8006 Zürich, Switzerland; ⊥ Institute of Food Chemistry, Leibniz University Hannover, Callinstraße 5, 30167 Hannover, Germany; # Faculty of Medicine, Saarland University, 66421 Homburg , Germany; g Center for Bioinformatics, Saarland University, 66123 Saarbrücken, Germany

## Abstract

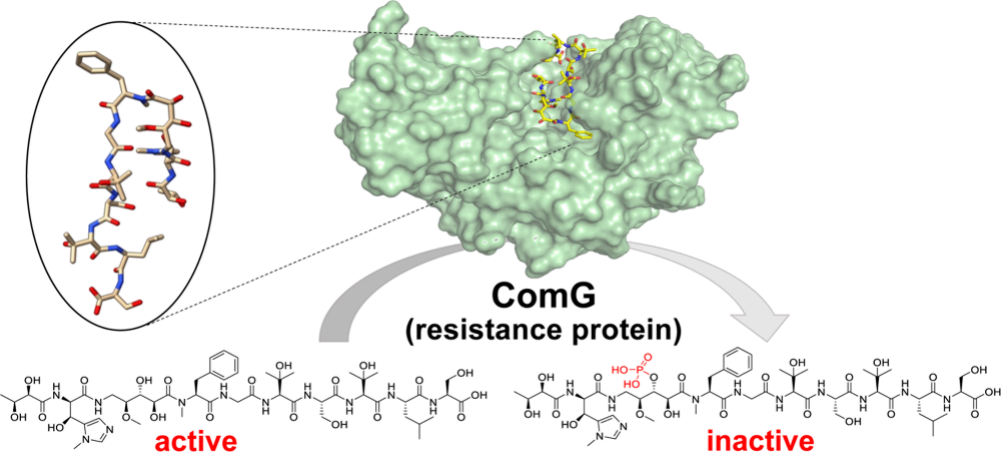

The rapid development of antibiotic resistance, especially among difficult-to-treat Gram-negative bacteria, is recognized as a serious and urgent threat to public health. The detection and characterization of novel resistance mechanisms are essential to better predict the spread and evolution of antibiotic resistance. Corramycin is a novel and modified peptidic antibiotic with activity against several Gram-negative pathogens. We demonstrate that the kinase ComG, part of the corramycin biosynthetic gene cluster, phosphorylates and thereby inactivates corramycin, leading to the resistance of the host. Remarkably, we found that the closest structural homologues of ComG are aminoglycoside phosphotransferases; however, ComG shows no activity toward this class of antibiotics. The crystal structure of ComG in complex with corramycin reveals that corramycin adopts a β-hairpin-like structure and allowed us to define the changes leading to a switch in substrate from sugar to peptide. Bioinformatic analyses suggest a limited occurrence of ComG-like proteins, which along with the absence of cross-resistance to clinically used drugs positions corramycin as an attractive antibiotic for further development.

## Introduction

The spread of antimicrobial resistance (AMR) represents one of the principal public health problems of this century and has consequently been declared as one of the top 10 global public health threats by the World Health Organization (WHO) in 2019.^1,2^ Infections with Gram-negative bacteria, in particular those included in the list of ESKAPE (*Enterococcus faecium*, *Staphylococcus aureus*, *Klebsiella pneumoniae*, *Acinetobacter baumannii*, *Pseudomonas aeruginosa*, *Enterobacter* spp.) pathogens, are a major concern due to their tendency to acquire multidrug resistance.^3,4^ Research efforts have thus largely focused on the development of new bioactive compounds against Gram-negative bacteria, ideally addressing innovative targets.^5,6^

**Figure 1:**
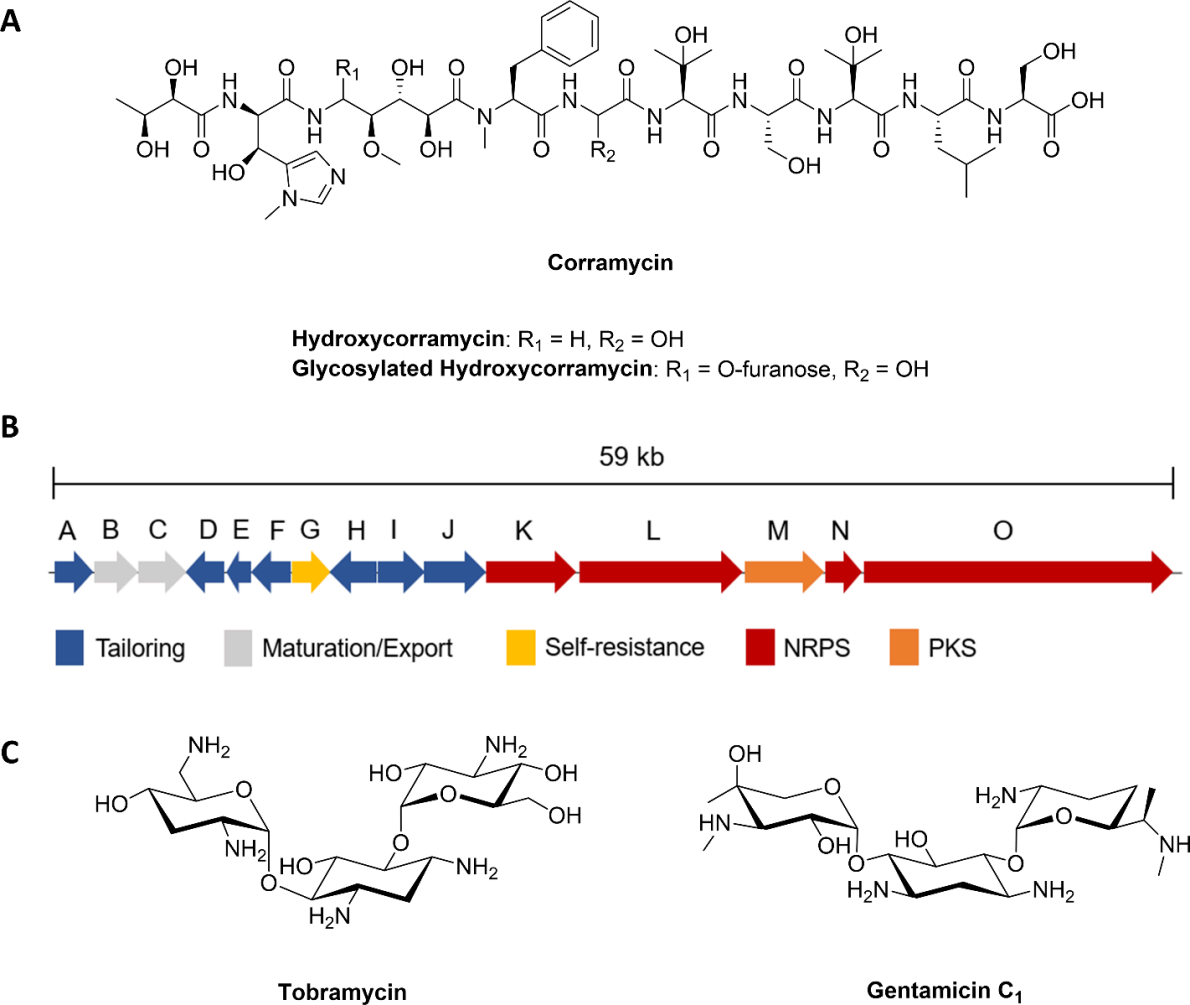
(A) Chemical structure of corramycins. (B) Representation of the biosynthetic gene cluster (BGC) of corramycins. Biosynthetic genes are colored based on their function. (C) Chemical structures of tobramycin (left panel) and gentamicin C_1_ (right panel). NRPS: nonribosomal peptide synthetase; PKS: polyketide synthase.

The investigation of the specialized metabolism of myxobacteria, δ-proteobacteria known for their complex life cycle and social behavior, has resulted in the isolation of natural products exhibiting high chemical diversity and unusual modes of action.^7^*Corallococcus coralloides* species are the producers of the natural products corramycins, which were isolated in an activity-guided isolation process aiming to identify novel chemical scaffolds exhibiting activity against Gram-negative pathogens. Corramycins (Figure 1A) are linear, peptidic natural products harboring several modified amino acids as well as an N-terminal butyric acid and a thus far undescribed γ-*N*-methyl-β-OH-histidine.^8,9^ The biosynthetic gene cluster (BGC) responsible for the production of corramycins was identified as a 12-modular hybrid nonribosomal peptide synthetase (NRPS)–polyketide synthase (PKS) megasynthetase assembly line organized in one operon (Figure 1B). The major product of this BGC exhibited substantial antimicrobial activity against different Gram-negative bacteria (e*.*g., minimum inhibitory concentration (MIC) against *Escherichia coli* NCTC13441 = 4 μg mL^–1^) and was active in a first *in vivo* proof of concept study in rodents.^8^ A hydroxylated derivative and a derivative with additional glycosylation were isolated as side products (Figure 1A). The mode of action of corramycins is still elusive, but given their potency and spectrum, we investigated how the producing organism had established self-resistance and whether the underlying resistance mechanism is already present among bacteria.

Genes related to self-resistance mechanisms being directly linked to the BGC encoding the biosynthesis is a common feature of microbial natural product production.^10^ This fact has even been exploited in the search for new natural products in the so-called self-resistance-guided genome mining.^11^ The understanding of the underlying mechanisms of bacterial resistance against antibiotics as well as their epidemiological distribution is vital to develop successful counterstrategies as early as possible in the drug development process. In general, the most common mechanisms of AMR by prokaryotes include enzymatic inactivation, target modification, porin defects, and overexpression of efflux transporters.^12,13^

Aminoglycosides, such as tobramycin and gentamicin (Figure 1C), are antibiotics of high clinical relevance due to their bactericidal activity and broad-spectrum profiles. Aminoglycoside phosphotransferases (APHs), which phosphorylate specific hydroxyl groups in aminoglycosides, are very effective inactivators of this class of antibiotics.^14,15^ Although other resistance mechanisms against aminoglycosides can occur, the synthesis of aminoglycoside-modifying enzymes (AMEs) is the predominant mode of resistance in clinical isolates. The evolutionary success of AMEs is attributed to the ability of most enzymes to act on more than one aminoglycoside in addition to their frequent occurrence on mobile genetic elements, increasing their likelihood of dissemination among species.^16^ Intriguingly, there have been scarce reports of enzymes with a substrate range beyond the aminoglycosides such as the kinase Cph, which confers self-resistance to the peptide-based antibiotic capreomycin.^17^ Capreomycin, viomycin, and derivatives thereof constitute the antibiotic class of tuberactinomycins, which represent effective treatments against multidrug-resistant tuberculosis (MDR TB).^18^ In another case, there are reports of low-level resistance to the structurally very different class of fluoroquinolones conferred by the acetylation of ciprofloxacin and norfloxacin by an enzyme related to an APH.^19,20^ Consequently, there have been hypotheses that aminoglycoside phosphotransferases and common kinases evolved from a mutual ancestor and share a general kinase fold and kinetic mechanism.^15^

Herein, we describe the kinase ComG from the corramycin BGC as a self-resistance factor of *C. coralloides*. We demonstrate that the expression of ComG leads to resistance in *E. coli*. The reconstitution of the ComG activity *in vitro* allowed us to pinpoint the position of phosphorylation on the corramycin backbone. The crystal structure of ComG in complex with corramycin revealed an unexpected β-hairpin-like conformation of the natural product and shed light on the catalytic mechanism. Importantly, ComG-like enzymes were not found widely distributed among bacteria, and related aminoglycoside-modifying enzymes do not confer resistance to corramycin, indicating little pre-existing resistance and, thus, showing great potential for corramycin to be developed as a novel class of antibiotics.

## Results and Discussion

### The Corramycin BGC Contains a Gene Related to Aminoglycoside Kinases

The corramycin BGC contains 15 genes, most of which belong to the modular hybrid PKS/NRPS assembly line and the corresponding tailoring genes for the biosynthesis of the natural product (Figure 1B). In the search for a characteristic self-resistance gene, we expressed several potential tailoring genes in *E. coli* and found *comG* to confer corramycin resistance (MIC 256 μg mL^–1^, Table S1).

A search of the nonredundant protein sequences database using blastP and ComG as a query revealed that its closest homologues are kinases conferring resistance to aminoglycoside antibiotics. We did not observe any shift in the inhibitory concentration of reference antibiotics, including aminoglycosides, for ComG-expressing *E. coli* (Table S1). Thus, ComG cannot confer resistance to members of this antibiotic class, suggesting rather poor structural homology between the substrate binding site of ComG and those of other aminoglycoside kinases. Based on this finding, we were intrigued to understand how the peptidic antibiotic corramycin may be bound and modified by ComG.

### ComG Phosphorylates Corramycin in an Unprecedented Position

**Figure 2:**
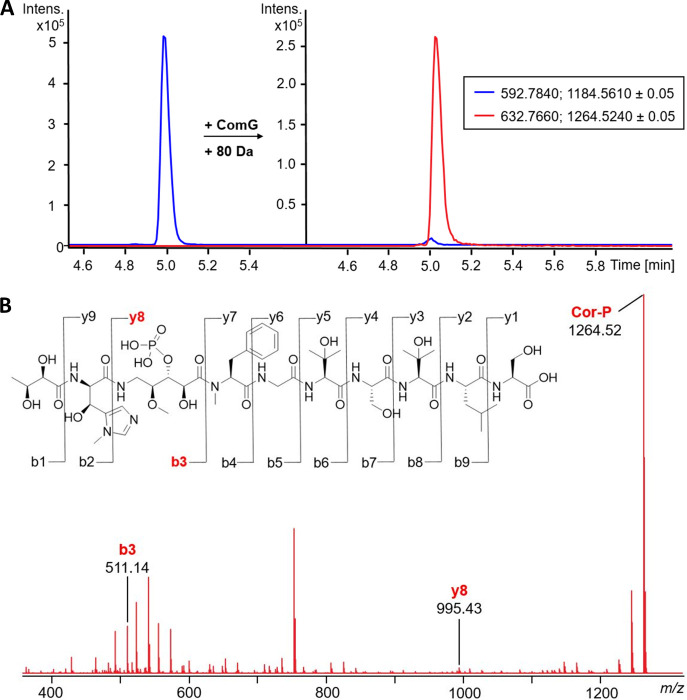
ComG phosphorylates corramycin in an unprecedented position. (A) HR-LCMS analysis of an *in vitro* reaction of corramycin with ComG and ATP/MgCl_2_. Extracted ion chromatograms (EIC) show a characteristic mass shift of +80 Da, hinting at the addition of a phosphate group (left panel: + ATP/– ComG; right panel: + ComG/ATP). The negative control depicting the reaction of corramycin with ComG, but without ATP, can be found in Figure S3. (B) Tandem MS/MS analysis of the product of the ComG reaction Cor-P (phosphorylated corramycin). Characteristic shifts of y8 and b3 ions highlight the position of phosphorylation being the β-alanine moiety. Full mass tables of corramycin and Cor-P fragmentation by MS/MS analysis are shown in Tables S2 and S3. NMR data of Cor-P can be found in Figure S7.

After we observed that ComG conferred resistance to *E. coli*, we attempted to reconstitute this activity *in vitro*. The enzyme was expressed in *E. coli* Lemo21 (DE3) and purified to homogeneity (Figure S2A), and analysis of ComG using intact protein mass spectrometry showed very good agreement between observed and calculated mass (*m*_obs_ = 40,949.37 Da, *m*_calc_ = 40,949.97 Da, Figure S2B). Upon incubation of corramycin with ComG in the presence of ATP and MgCl_2_ at 22 °C, we observed a mass shift of +79.97 Da, corresponding to the addition of a phosphate group (Figure 2A). As expected, no substrate conversion was observed without the addition of ATP (Figure S3). Interestingly, ComG heavily favors ATP over GTP as the phosphate donor for its catalytic function (Figure S4), which is in contrast to the capreomycin kinase Cph, which shows comparable conversion for both nucleotides.^17^ In addition, the enzyme kinetics for the reaction of ComG with ATP were analyzed for a series of corramycin concentrations, and *K*_m_ and *k*_cat_ values were determined to be 33 μM and 1 min^–1^, respectively (Figure S5).

We hypothesized that the phosphorylation of corramycins may be the inactivation mechanism inside living cells. To support this hypothesis, the inhibitory concentration of the purified, phosphorylated corramycin (Cor-P) against *E. coli* was determined. Cor-P showed no antibacterial activity (inhibitory concentration >64 μg mL^–1^), confirming the inactivation of corramycin via phosphorylation by ComG. To identify the site of phosphorylation, we analyzed Cor-P using tandem mass spectrometry (MS^2^) and nuclear magnetic resonance (NMR). MS^2^ of Cor-P confirmed a mass shift of +80 Da and placed the mass shift on the characteristic b3 and y8 ions (Figures 2B and S6, Tables S2 and S3). Superposition of the HMBC NMR spectra of corramycin and Cor-P showed characteristic ^1^H- and ^13^C-shifts of 0.3 ppm and approximately 3 ppm, respectively, for the C_3_ atom of the β-alanine incorporated by the PKS of the BGC. These data agree with the site of modification as determined by MS^2^ and allowed us to pinpoint the specific atom that is phosphorylated (Figure S7).

### Overall Structural Analysis of ComG

Aminoglycoside kinases have been the subject of intense research due to their involvement in AMR against an important clinical class of antibiotics. As aminoglycosides and the linear peptide corramycin do not share obvious chemical or structural space, we were intrigued by the idea of obtaining structural information on ComG. Low sequence identity to published aminoglycoside phosphotransferase protein structures required us to produce ComG in which methionine had been replaced by selenomethionine for experimental phasing. This protein crystallized in space group *P*2_1_, and a data set was collected to a resolution of 2.1 Å. The structure was determined using single-wavelength anomalous dispersion (all data collection and refinement statistics can be found in Table S4). The presence of translational pseudosymmetry complicated refinement of the structure.

**Figure 3:**
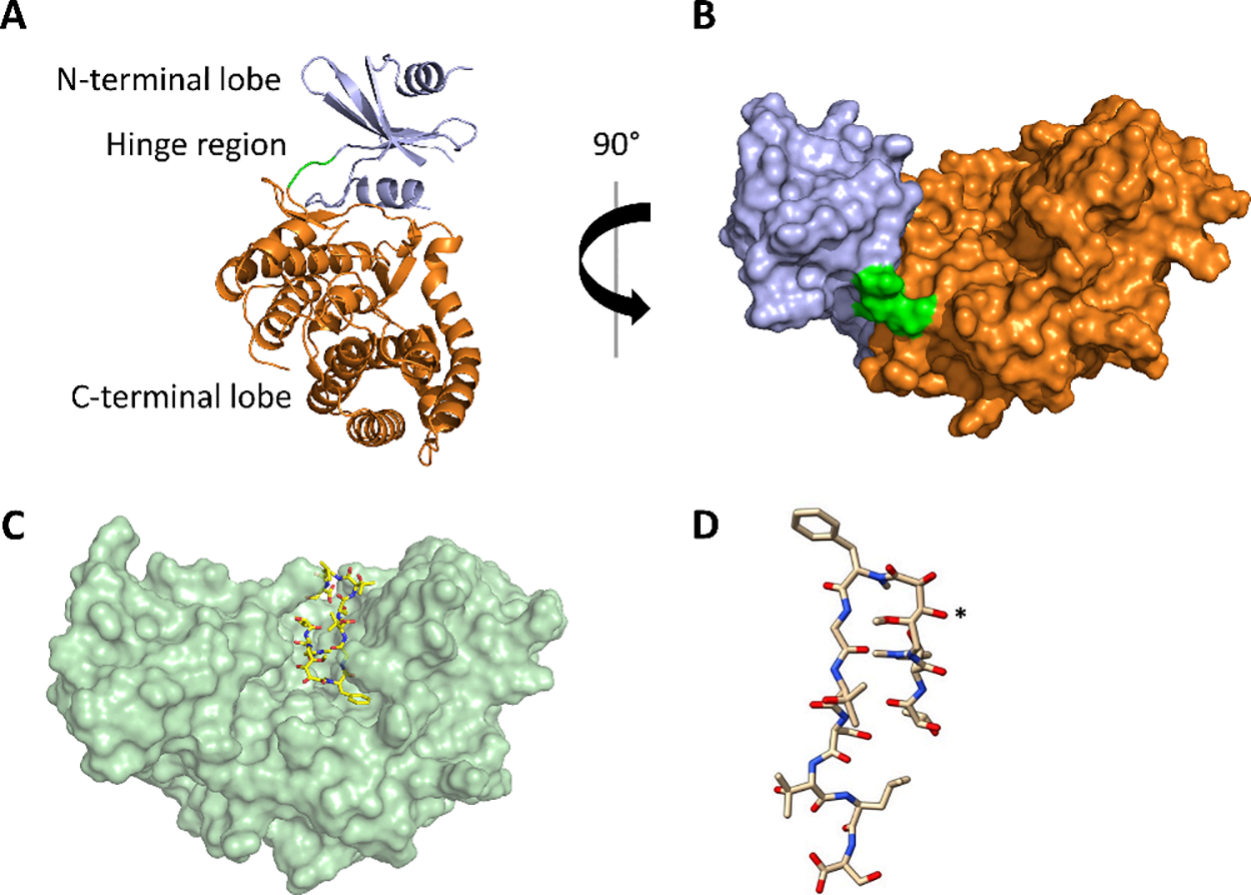
(A) Cartoon representation of the crystal structure of apo ComG, highlighting the typical bilobed kinase fold. The N-terminal lobe is shown in blue, the hinge region in green, and the C-terminal lobe in orange. (B) Surface representation of the crystal structure of apo ComG, with the same color coding as that in A. (C) Surface representation of ComG^C^. Protein residues are shown in pale green, whereas corramycin is shown as yellow sticks. (D) Structure of corramycin bound to ComG adopts a β-hairpin-like structure. The hydroxyl group that is phosphorylated by ComG is marked with an asterisk.

Apo ComG contained two protomers in the asymmetric unit, and the refined model encompassed residues 14–33, 39–57, and 66–364 for chain A (13–33, 40–56, and 66–364 for chain B). The enzyme adopts the typical bilobed kinase fold, with the N-terminal lobe being formed primarily by β-sheets, while the C-terminal lobe consists predominantly of α-helices (Figure 3A). The ATP-binding hinge region is found in the loop between the two lobes, with the conserved glycine residue leading to the parallel orientation of the backbone, which in turn is expected to coordinate the adenine of ATP. On the opposing side of the hinge region lies the “DFG loop”, which in ComG is mutated to a DFE motif (Figure S8). The conserved aspartate residue is critical for the coordination of the magnesium cation and, thus, for the orientation of the phosphate region of ATP. The surface representation of ComG revealed a wide pocket between the N- and C-terminal lobes that appeared suitable to accommodate a larger natural product (Figure 3B). In addition, residues on both sides of the pocket extending toward the solvent could possibly act as a closing lid above both the substrate and cofactor binding sites.

A search for structural homologues of ComG using the DALI server revealed an uncharacterized kinase (PDB ID 3csv) as the closest hit with a C_α_ RMSD of 4.0 Å and 79% sequence coverage. The closest structural homologue for which the structure had been determined with a bound substrate was aminoglycoside 7″-phosphotransferase-Ia, which phosphorylates and thereby inactivates the aminoglycoside antibiotic hygromycin B (PDB ID 6IY9) (Figure S9). The overall structural homology is fair, with a C_α_ RMSD of 3.73 Å, but only just over half of the C_α_ atoms could be aligned structurally (52%). The poor structural homology was somewhat expected, since ComG acts on a peptidic substrate and led us to pursue crystallization of the ComG–corramycin complex.

### Corramycin Adopts a β-Hairpin-like Structure

To this end, ComG was incubated with a 5-fold molar excess of corramycin, a 10-fold molar excess of the nonhydrolyzable ATP analogue AMPPCP, and MgCl_2_ for 24 h at 4 °C before crystallization trials were attempted. ComG complex crystals (ComG^C^) were observed in multiple conditions and also belonged to space group *P*2_1_, and a data set was processed to a resolution of 1.5 Å. The structure of ComG^C^ was determined by molecular replacement using the ComG structure as a search model. Interestingly, the addition of corramycin led to a significant difference in crystal packing, as ComG^C^ only had a single protomer in the asymmetric unit, and the pathology observed for ComG crystals was no longer present. Binding of corramycin did not lead to notable movement in the structure of ComG when comparing apo and complex structures (C_α_ RMSD of 0.35 Å), with the exception of the loop connecting residues 33 and 39, which is ordered in the ComG^C^ structure. This is likely a direct result of a hydrogen bond between corramycin and Arg38 (Figure S10).

We observed unambiguous electron density for corramycin in a pocket formed between the N- and C-terminal lobes but could not observe any density for AMPPCP/Mg^2+^ (Figures 3C and S10). Unexpectedly, the three-dimensional structure of corramycin was reminiscent of two antiparallel β-strands, which adopt a β-hairpin-like structure that is stabilized by six intramolecular hydrogen bonds (Figures 3D and S11). The turn of the hairpin is composed of the *N*-methyl-phenylalanine, the side chain of which is bound in a hydrophobic pocket, and part of the hydroxylated γ-amino valeroyl moiety, which is also the site of corramycin phosphorylation by ComG. This region of corramycin is further stabilized by two hydrogen bonds between the natural product and ComG (Figure S12). Interestingly, the position of this moiety at the turn, the site of phosphorylation, leads to a conformation that could be viewed as mimicking a hexose (Figure 3D), with both hydroxyl groups pointing toward the putative ATP-binding site.

Structures of APH-related enzymes in complex with peptide-based natural products have been reported only once, for the capreomycin kinase Cph.^17^ Even though the bilobed architecture of both enzymes is somewhat similar, the overall structural homology is low (C_α_ RMSD of 6.22 Å) and, thus, even lower than the homology to the more unrelated APHs (Figure S13). The biggest structural differences are the absence of two C-terminal ComG loops and one α-helix (residues 285–315 and 345–364) in Cph, which are crucial for the formation of the hydrophobic pocket and, therefore, for corramycin binding. In contrast, the characteristic macrocycle of capreomycin is extending into the helical architecture of the C-terminal lobe, leading to the compound adopting a “C” shape.^17^ Intriguingly, this conformation is also the result of multiple intramolecular hydrogen bonds. Somewhat expected based on these results, we were unable to detect any phosphorylation of capreomycin by ComG, which highlights the structural differences of the substrate binding pocket (Figure S14 and Table S1).

### Modeling of the Catalytic Center

**Figure 4:**
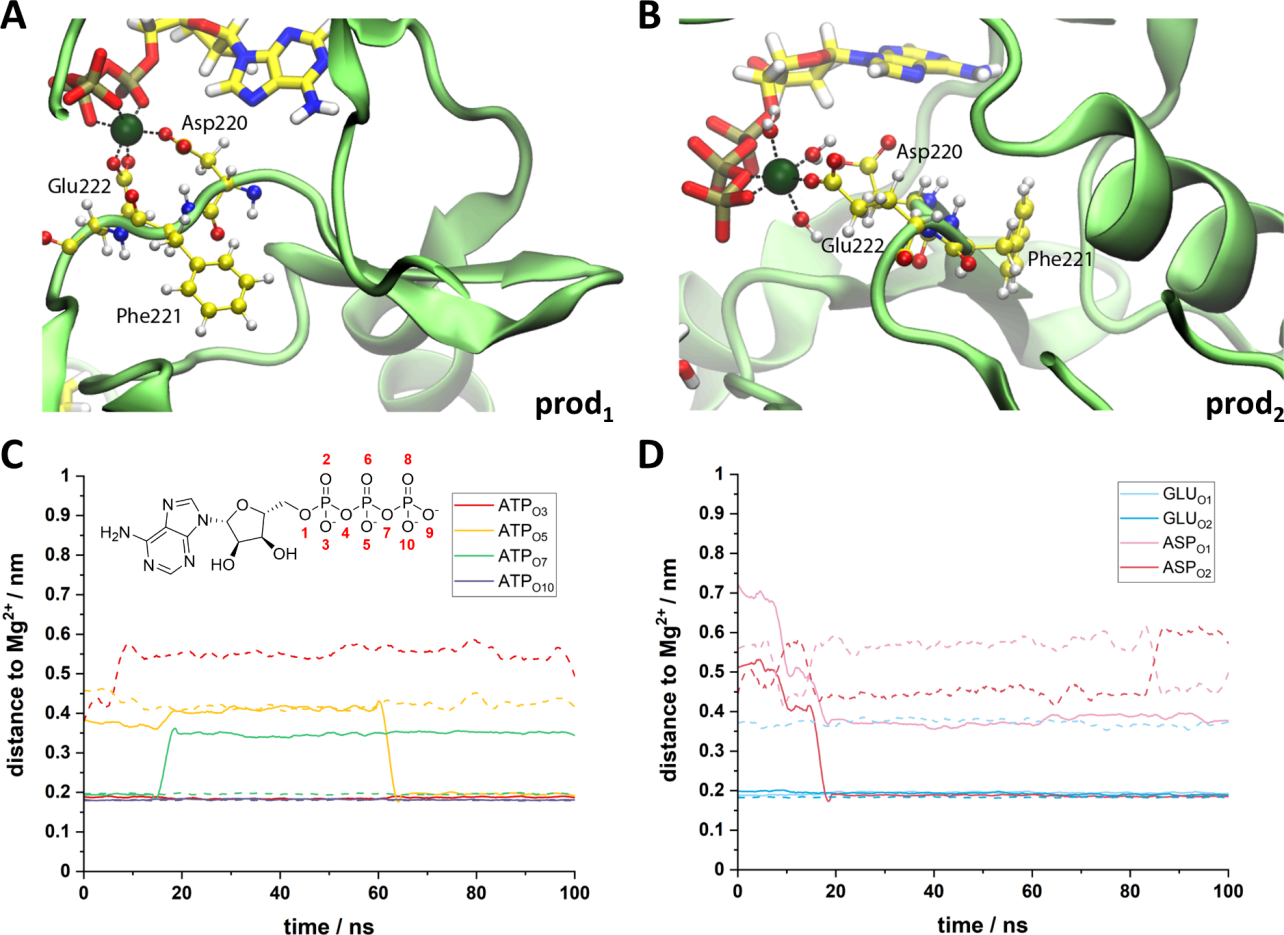
Mg^2+^ coordination center. (A, B) Representation of the coordination center in production 1 (A) and production 2 (B). Mg^2+^ is shown in dark green, DFE residues and water are shown with balls and sticks, and ATP is shown with sticks. Carbon atoms are colored in yellow, nitrogen in blue, oxygen in red, phosphorus in gold, and hydrogens in white. Protein backbone is shown in pale green. (C, D) Distance between the Mg^2+^ center and the oxygen ligands from ATP (C) and from Asp220 or Glu222 (D). Results from production 1 are shown with solid lines, and results from production 2 are shown with dashed lines. Oxygens from ATP are labeled as shown in the representation on the upper left side of C.

Despite adding a 10-fold molar excess of AMPPCP/MgCl_2_, we did not observe convincing electron density for the ATP analogue in the complex structure. This may be due to a rather mediocre affinity or the fact that corramycin binding in the active site with a high affinity occluded AMPPCP from the pocket during the crystallization process. To better understand the catalytic mechanism, we set out to model ATP into our complex structure using molecular dynamics (MD) simulations. As an initial step, one ATP and one Mg^2+^ were placed at the presumed ATP-binding site of ComG (hinge region, DFE motif). Next, the system was minimized, equilibrated, and simulated for 100 ns. In our simulations, the adenine group of ATP established an H-bond with the backbone carbonyl oxygen of Leu100 from the hinge region (Figure S15). The Mg^2+^ center is hexacoordinated with an octahedral geometry, the most usual coordination for Mg^2+^ in biological macromolecules,^21^ which was previously described for an aminoglycoside kinase.^22^ According to our simulations, Mg^2+^ is coordinated by two oxygens of the side chains of Asp220 and Glu222 from the DFE loop of ComG and by the triphosphate group of ATP (Figure 4). Despite ATP acting as a ligand in both of the simulations, in one of them, it acted as a tridentate ligand (production 1) and in the other as a bidentate ligand (production 2, Figure 4C). The coordination with Glu222 is very stable and was observed in both of our simulations; Asp220, however, acted as a ligand in only one of our simulations (production 1, Figure 4D).

Furthermore, we analyzed the cofactor binding preferences of ComG by comparing the modeled ATP pose to that of bound nucleotides in other APHs (Pfam family PF01636, Table S6). Although sequence conservation in this family is low, the amino acid pattern in ComG indicates a larger similarity to the ATP binding pocket in other kinases (Figure S16).

The role of the water molecules in the coordination of the Mg^2+^ center is also important, at least as a transient ligand. In fact, in one of our simulations (production 2), Mg^2+^ is coordinated with three water molecules for the full 100 ns of our simulation (Figure 4B). Furthermore, the orientation of the N-terminal chain of corramycin also seems to be important for its interaction with ATP, as depicted in the comparison between the position of corramycin after 90 ns of simulation in productions 1 and 2 (Figure S17). We also followed the distance between the oxygen of the C_3_-hydroxyl group derived from the carboxy group of β-alanine from corramycin and the terminal phosphate from ATP along the simulated time (Figure S18), and all simulation data agree with experimental observation.

### Bioinformatic Analysis and Evolution of ComG

Naturally occurring resistance is a major concern in the exploration of novel potential antibiotics. We thus performed a thorough bioinformatic analysis to explore the evolutionary spread of potential resistance mediated by ComG-like proteins through genome mining in the order *Myxococcales* and the phylum *Actinobacteria* (Figure S19). In the *Myxococcales*, we only found one homologue with high sequence identity on the protein level (72.5%, Figure S1) in three identical NRPS/T1PKS-type clusters in *Myxococcus llanfairpwllgwyngyllgogerychwyrndrobwllllantysiliogogogochensis*, *Myxococcus guangdongensis*, and *Myxococcus* sp. CA040A (Figure S19). In *Actinobacteria*, we found several homologues with a relatively low sequence identity (25–30%) but good coverage (sequence similarity observed over >75% of the entire ComG length) in BGCs of very different types, ranging from aryl polyene to NRPS and siderophores. These data may indicate other instances in which aminoglycoside-like phosphotransferases acquired a substrate range that is broader than aminoglycoside antibiotics, but experimental verification will be required before firm conclusions can be drawn.

Our bioinformatic analysis, along with the structural data presented above, supports the notion that aminoglycoside-like kinases are branched in the kinase protein family and share an evolutionary origin as well as catalytic mechanisms with other kinases.

**Figure 5:**
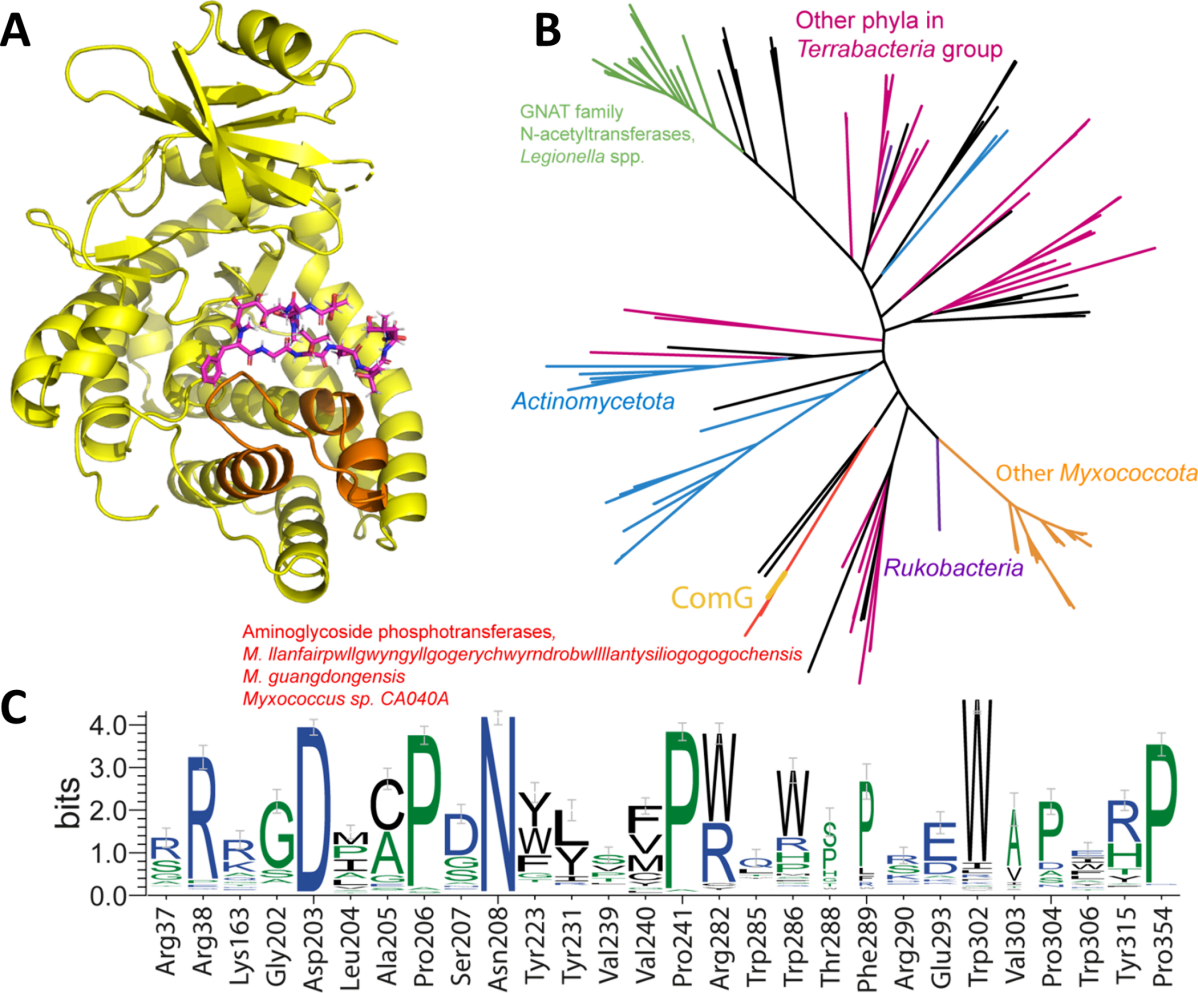
(A) Cartoon structure of ComG. The C-terminal corramycin-binding region between Trp285 and Tyr315 is colored orange, whereas the natural product is shown as violet sticks. (B) Phylogenetic tree of ComG-like proteins with a conserved C-terminal corramycin-binding region. Branches corresponding to important taxa are shown in color. ComG is shown in gold. (C) Sequence logo of conservation of corramycin-binding residues.

In order to further characterize potential pre-existing corramycin resistance in bacteria, we explored the phylogenetic spread of ComG-like enzymes, irrespective of their position in BGCs. ComG belongs to the phosphotransferase enzyme family APH (Pfam family PF01636). However, this domain does not extend to the C-terminal region of the protein, comprising positions 285–315 (Figure 5A), which is crucial for corramycin binding. Thus, most proteins in this family are likely incapable of binding corramycin.

To narrow down the potential set of corramycin-binding APHs, we searched for homologues of ComG with BLAST and filtered them in such a way that the sequence similarity extended into the corramycin-binding C-terminal region. This yielded 202 proteins, predominantly phosphotransferases from *Myxococcota* and *Actinomycetota* and kinases and hypothetical proteins in other phyla from the *Terrabacteria* group such as *Armatimonada*, *Bacillota*, *Cyanobacteriota*, and *Chloroflexota*, as well as from a candidate phylum *Rokubacteria*. The corramycin kinase lies on a branch separated from other myxobacteria, with four proteins from *Myxococcus* sp. CA040A, *M. llanfairpwllgwyngyllgogerychwyrndrobwllllantysiliogogogochensis*, and *M. guangdongensis* that are annotated as phosphotransferases or aminoglycoside phosphotransferase family proteins. In these species, the ComG homologue is embedded in a BGC that is very similar to the corramycin BGC. A distant branch corresponds to GNAT family *N*-acetyltransferases from *Legionella* spp. and several other closely related proteins from *Pseudomonadota* (Figure 5B). Analysis of conservation of corramycin-binding residues in these proteins shows a reasonable conservation within the phosphotransferase domain but a severe drop of conservation in the corramycin-binding C-terminal region, spanning positions 285–315 (Figure 5C). Structural analysis shows that this region is crucial for binding of corramycin. These data indicate that ComG is a distinct kinase with very few homologues in the kingdom of bacteria.

In order to identify potential naturally occurring resistance in pathogenic bacteria, we searched the CARD database (Comprehensive Antibiotic Resistance Database^23^) for homologues of ComG, but in none of these hits did homology expand to the corramycin-binding C-terminal part. Together with an overall low homology, we propose that these proteins do not confer corramycin resistance. When we further analyzed the ESKAPE pathogens, we found five hits that were deemed significant and fell within the CARD branch of the phylogenetic tree (Figure 5B). Based on their taxonomic diversity, three genes were cloned, expressed in *E. coli* BL21 (DE3) (Figure S20), and tested for their activity toward corramycin. As expected and unlike ComG, we observed no MIC shift for the three tested ComG homologues, and they thus do not confer resistance to corramycin (Table S7).

### Corramycin Overcomes Aminoglycoside Resistance Mediated through AMEs

**Figure 6:**
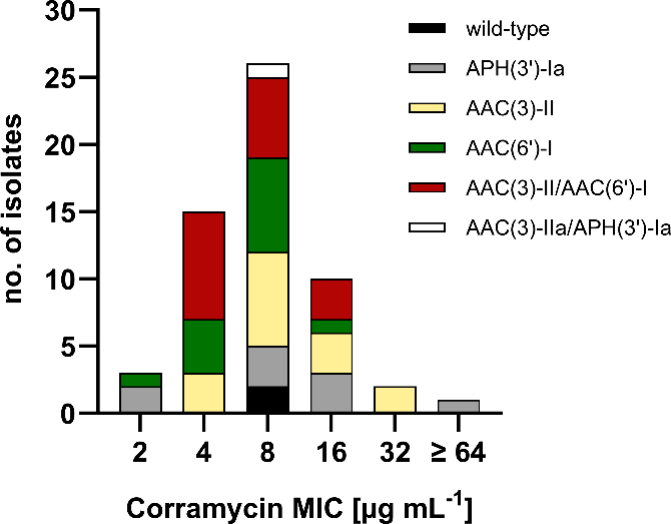
Distribution of corramycin MICs among 57 multidrug-resistant *Escherichia coli* clinical isolates. Corramycin is not affected by aminoglycoside-modifying enzymes (AMEs), irrespective of the class of AMEs. APH: aminoglycoside phosphotransferase; AAC: aminoglycoside acetyltransferase; no.: number.

Given the relation of ComG to aminoglycoside phosphotransferases, we aimed to investigate a potential cross-resistance to this class of antibiotics. To this end, we assessed the antibacterial activity of corramycin against 57 *E. coli* clinical isolates, 55 of which express different classes and combinations of AMEs (Table S8).^24^ Corramycin MICs were distributed unimodally with an MIC_50_ of 8 μg mL^–1^ and MIC_90_ of 16 μg mL^–1^ (Table 1, Figure 6). Besides imipenem and colistin, corramycin was the only antibiotic that remained active, irrespective of the resistance mechanism, indicating that the peptide is not inactivated by AMEs nor is it affected by other antimicrobial resistance determinants conferring resistance to antibiotics like fluoroquinolones, tetracyclines, or trimethoprim.

**Table 1:** MIC_50_ and MIC_90_ of Corramycin and Reference Antibiotics among 57 Multidrug-Resistant *Escherichia coli* Clinical Isolates^a^

		MIC [μg mL^–1^]		
antibiotic	EUCAST CBP^25^ [μg mL^–1^]	MIC range (*n* = 57)	MIC_50_	MIC_90_
corramycin	n/a	2 to >64	8	16
amikacin	8	4 to 64	16	32
gentamicin^b^	2	2 to >64	>64	>64
kanamycin^c^	n/a	4 to >64	>64	>64
neomycin^d^	n/a	2 to >64	4	>64
tobramycin	2	2 to >64	32	>64
imipenem	2	0.25 to 2	0.5	1
colistin	2	0.25 to 0.5	0.5	0.5
ciprofloxacin	0.25^e^	0.016 to >8	>8	>8
levofloxacin	0.5	0.03 to >16	16	>16
tetracycline	n/a	2 to >64	>64	>64
trimethoprim	4^f^	0.5 to >64	>64	>64

a Minimum inhibitory concentrations (MICs) were determined in cation-adjusted Mueller-Hinton broth (MHB2) using the broth microdilution method according to the EUCAST guidelines. Values represent two independent repeats. CBP: clinical breakpoint; EUCAST: European Committee on Antimicrobial Susceptibility Testing; n/a: not available.

b Combination of gentamicin C_1_, C_1a_, and C_2_.

c Main component kanamycin A.

d Main component neomycin B.

e Indications other than meningitis.

f Uncomplicated urinary tract infections (UTI).

Remarkably, one of the clinical isolates expressing APH(3′)-Ia displayed resistance to corramycin (MIC > 64 μg mL^–1^). Nevertheless, we can exclude the involvement of the phosphotransferase, as corramycin maintained activity against other isolates expressing the same APH variant. The majority of the tested isolates are multidrug-resistant (Table 1, Table S9); thus, it appears likely that the loss of susceptibility is caused by a distinct mechanism. To identify the culprit of resistance, we carefully reassessed the genome of the isolate with a particular focus on the *sbmA* gene. We have previously described that mutations of SbmA, a transporter responsible for the uptake of the antibiotic in *E. coli*, can confer resistance to corramycin.^8^ Strikingly, we found a point mutation in the *sbmA* gene resulting in a premature stop codon and, hence, a truncated protein. We further assessed the susceptibility of the uptake-deficient strain in M9 minimal medium and found that corramycin activity was restored with an MIC of 1 μg mL^–1^. This result supports our hypothesis of SbmA inactivation being the root of resistance in *E. coli*, as corramycin uses an additional uptake system under minimal conditions and does not solely rely on SbmA. We have previously demonstrated that single SbmA mutations, despite conferring resistance *in vitro*, may not necessarily translate into a loss of efficacy *in vivo*.^8^ These findings, coupled with the absence of cross-resistance to clinically used drugs, highlight the potential of corramycin in overcoming drug resistance.

## Conclusion

The in-depth study of antibiotic self-resistance mechanisms plays a vital role in controlling the spread of AMR and its associated problems for human health. This is especially true for compounds active against Gram-negative bacteria, as infections caused by such pathogens are generally more difficult to cure due to limited treatment options. Novel antibiotics with innovative targets are needed. The development of such is, however, time-consuming and expensive,^26,27^ which makes the early, thorough understanding of resistance mechanisms and their spread among pathogenic bacteria a very high priority.

Besides self-resistance mechanisms of tuberactinomycins,^17^ ComG is the first kinase within the APH enzyme family able to modify and inactivate a peptidic antibiotic. Unlike the capreomycin kinase Cph, however, ComG shows cofactor specificity toward ATP and adopts a unique 3D-fold tailored toward corramycin binding. It was particularly surprising to discover that corramycin adopts a β-hairpin-like structure, which in turn allows the part of the molecule that is modified by ComG to mimic a sugar. It is unclear whether the active conformation of corramycin, or indeed its solution structure, reflects that of corramycin bound to ComG. Stabilization of the conformation observed in the ComG^C^ crystal structure by six hydrogen bonds, combined with the presence of the α-*N*-methylated phenylalanine at the β-hairpin turn, may be sufficient to stabilize the conformation in solution. The presence of a special “C”-shaped conformation of capreomycin, which is also the result of intramolecular hydrogen bonds, may hint at an evolutionary design for peptide antibiotics. Corramycin appears to combine two very important areas of medicinal chemistry in one molecule: β-hairpins and carbohydrate-mimetic peptides. It may therefore serve as a valuable starting point for future synthetic efforts to develop bioactive molecules featuring either one or both elements.

The effect of corramycin phosphorylation by ComG on target engagement by the antibiotic remains elusive, but we view it as probable that, analogously to aminoglycoside antibiotics, binding to the target is abolished by phosphorylation. A potential mode of dephosphorylation as activation mechanism has yet to be analyzed, but without a suitable gene product in the corramycin BGC, it may either occur spontaneously or, more likely, be the result of generic phosphatases. It remains unclear whether phosphorylation is the main way by which the producing strain protects itself against corramycin. We have previously hypothesized that corramycin may be produced as a fatty acid-linked pro-drug that is exported and subsequently hydrolyzed in the periplasm similar to the description of the myxobacterial natural product vioprolide.^8,28^ Thus, ComG might only function as a highly efficient fail-safe in case of a “production error” or unintentional import of released corramycin, ultimately eliminating the need for a reactivation mechanism.

Since phosphorylation is a very effective way to neutralize antibiotics, we performed genome mining studies to analyze the spread of potential ComG homologues. The results were very promising in that the spread is limited and mostly restricted to predicted biosynthetic gene clusters. Critically, no presence on mobile genetic elements could be determined, and our data indicate a rather narrow substrate range of ComG with no activity detected on aminoglycosides, macrolides, fluoroquinolones, and the peptide antibiotic capreomycin. We further characterized the activity of corramycin against multidrug-resistant *E. coli* clinical isolates expressing different types of AMEs including common aminoglycoside phosphotransferases. No cross-resistance was observed, suggesting that the natural product is not part of the substrate range of AMEs. To adapt the highly prevalent aminoglycoside phosphotransferases for activity on corramycin would, among extensive mutations, also require a specific C-terminal extension of these proteins. We are of the opinion that such a process would likely be very slow, which, in conjunction with its intriguing structure, the lack of cross-resistance with commercially used antibiotics, and a most likely innovative target renders corramycin a very promising antibiotic natural product primed for further development.

## Supporting Information Available

Experimental details, materials, and methods, as well as supplementary figures and tables (PDF)

## Accession Codes

Diffraction data and refined structural models have been deposited in the PDB. See Table S4 for PDB codes.

## ABBREVIATIONS

GNAT: GCN5-related *N*-acetyltransferase
HMBC: heteronuclear multiple bond correlation
RMSD: root mean square deviation
