# Supplementary material for: The Peptide Antibiotic Corramycin Adopts a β-Hairpin-like Structure and Is Inactivated by the Kinase ComG
Source: J Am Chem Soc. 2024 Mar 21;146(13):8981–90. doi: 10.1021/jacs.3c13208 (PMC10996006; doi:10.1021/jacs.3c13208)
Supplement: Supplementary file 1 — ja3c13208_si_001.pdf [file ja3c13208_si_001.pdf]

The Peptide Antibiotic Corramycin Adopts a ß-hairpin-like Structure and is Inactivated by the Kinase ComG

Sebastian Adam[a]‡, Franziska Fries[a,b,c]‡, Alexander von Tesmar[a]‡, Sari Rasheed[a,c], Selina Deckarm[a], Carla F. Sousa[a], Roman Reberšek[a], Timo Risch[a,b], Stefano Mancini[d], Jennifer Herrmann[a,c], Jesko Koehnke[a,e], Olga V. Kalinina[a,f,g], and Rolf Müller[a,b,c]*

[a] Helmholtz Institute for Pharmaceutical Research Saarland (HIPS), Helmholtz Centre for Infection Research (HZI), Saarland University, Campus E8 1, 66123 Saarbrücken, Germany

[b] Department of Pharmacy, Saarland University, 66123 Saarbrücken, Germany

[c] German Center for Infection Research (DZIF), Partner Site Hannover-Braunschweig, 38124 Braunschweig, Germany

[d] Institute of Medical Microbiology, University of Zürich, 8006 Zürich, Switzerland

[e] Institute of Food Chemistry, Leibniz University Hannover, Callinstr. 5, 30167 Hannover, Germany

[f] Faculty of Medicine, Saarland University, Homburg, Germany

[g] Center for Bioinformatics, Saarland University, Saarbrücken, Germany.

‡ These authors have contributed equally.

Table of Contents

Experimental Procedures 4

Strains....................................4

Cloning of *comG*....................................4

Construction of ComG and ComG homologues in *E. coli* 4

Resistance validation....................................5

Antimicrobial susceptibility testing of clinical isolates 5

Large scale protein expression of ComG 6

Selenomethionine expression of ComG 6

Protein purification of ComG 7

Crystallization of ComG....................................8

Data processing, structure determination, refinement and structural analysis of ComG......8

ComG *in vitro* assay 8

Purification, resistance validation and structure determination of Cor-P 10

Molecular dynamics (MD) simulations....................................10

Bioinformatics analysis 11

Supplementary Figures 12

Figure S1. 12

Figure S2 13

Figure S3 14

Figure S4 15

Figure S5 16

Figure S6 17

Figure S7 18

Figure S8 19

Figure S9 20

Figure S10 21

Figure S11 22

Figure S12 23

Figure S13 24
Figure S14. 25
Figure S15 26
Figure S16 27
Figure S17 28
Figure S18 29
Figure S19 30
Figure S20 31
Supplementary Tables....................32
Table S1 32
Table S2 33
Table S3 33
Table S4 34
Table S5 34
Table S6 35
Table S7 36
Table S8 37
Table S9 38

Experimental Procedures

Strains

Cloning was carried out in *E. coli* DH10β or HS996. Protein expression was conducted in *E. coli* Lemo21 (DE3) or in *E. coli* BL21 (DE3). Genomic DNA of *Corallococcus coralloides* ST201330 was used as template for PCR amplification.

Cloning of *comG*

The corresponding primers for every construct were ordered from Sigma-Aldrich (Table S5) and the different gene constructs amplified by PCR with a standard Phusion polymerase protocol with adjusted annealing temperatures. For the ComG construct with an additional N-terminal TEV site, the TEV recognition sequence was introduced with an extended primer right after the *Nde*I restriction site in pET28b+. DNA amplificates were subsequently digested with *Nde*I and *Hind*III in buffer O at 37 °C for 4 h and ligated into pET28b+ vector cut with the same enzymes and treated with alkaline phosphatase with a standard T4 DNA ligase protocol. Ligations were transformed into chemically competent *E. coli* DH10β or HS996 cells with a standard heat shock protocol, plated on Luria-Bertani (LB) agar with added 50 µg/mL kanamycin and grown at 37 °C for 16 h. Single *E. coli* colonies were grown in 10 mL LB-medium with 50 µg/mL kanamycin for 16 h at 37 °C and 200 rpm. 4 mL of grown cultures were centrifuged for 10 min at 4000 rpm and 4 °C and the cell pellet was used for a plasmid preparation by alkaline lysis. Extracted plasmids were digested with the respective restriction enzymes used for cloning using the same procedure as above and analyzed by agarose gel electrophoresis. Plasmids, which were found to carry the correct insert, were sent for sequencing by LGC genomics (Berlin) and subsequently transformed into chemically competent *E. coli* Lemo21 (DE3) cells.

Construction of ComG and ComG homologues in *E. coli*

Three *E. coli* codon optimized ComG homologous sequences with an N-terminal 6x His-tag, inserted in pET28b+, as well as ComG with an N-terminal 6x His-tag, inserted in pET19b, were ordered from a gene synthesis company (BioCat GmbH). The bacterial protein expression strain *E. coli* BL21 (DE3), previously transformed with the expression vector (according to standard protocols[1]), was cultivated for 16 to 24 h in LB medium at 28 °C. Protein expression was induced by adding 0.1 mM IPTG at an OD_{600} of 0.7. The cell pellet was harvested, resuspended in lysis buffer (150 mM NaCl, 20 mM imidazole, 25 mM Tris-HCl, pH 7.5), sonicated and centrifuged. The supernatant was used to perform a pull-down assay in a

magnetic particle processor according to manufacturer protocols (Thermo Scientific KingFisher mL Purification Syst. Magnetic Particle) and protein expression was verified by SDS-PAGE (Figure S20).

Resistance validation

Cultures of *E. coli* BL21 (DE3) expressing ComG and ComG homologues as well as the wild-type (previously transformed with the empty expression vector) were prepared in LB supplemented with 50 µg/mL kanamycin (pET28b+) or 100 µg/mL ampicillin (pET19b) and incubated overnight (30 °C, 300 rpm). Cultures were diluted 1:10 with fresh LB (supplemented with 50 µg/mL kanamycin or 100 µg/mL ampicillin) and incubated for 1 h (30 °C, 300 rpm). Protein expression was induced by addition of 0.1 mM IPTG and incubation for additional 1 h (30 °C, 300 rpm). Serial dilutions of corramycin and reference antibiotics were prepared in a 96-well plate in broth supplemented with 50 µg/mL kanamycin or 100 µg/mL ampicillin and 0.1 mM IPTG. Optical density (OD_{600}) of bacterial cultures was adjusted to a final cell count of ~$1x10^6$ CFU/mL. The plates were incubated for 18 h (30 °C, 300 rpm) and the inhibitory concentration was defined as the lowest concentration causing complete inhibition of visible growth. Erythromycin and kanamycin (main component kanamycin A) were obtained from Carl Roth (Karlsruhe, Germany). Amikacin, ciprofloxacin, clarithromycin, gentamicin (combination of gentamicin C_1, C_{1a} and C_2), levofloxacin, neomycin (main component neomycin B) and tobramycin were obtained from Sigma-Aldrich (St. Louis, MO, USA). Capreomycin was kindly provided by the National Reference Center for Mycobacteria (NRZ, Borstel, Germany).

Antimicrobial susceptibility testing of clinical isolates

E. coli clinical isolates were collected in and provided by the Institute of Medical Microbiology (IMM), University of Zürich. Two susceptible (wild-type) strains and 55 strains classified as resistant to at least one aminoglycoside (presence of AME-encoding genes confirmed by whole genome sequencing, Table S8) were used in this study[2]. Kanamycin (main component kanamycin A) was obtained from Carl Roth (Karlsruhe, Germany). Amikacin, ciprofloxacin, colistin, gentamicin (combination of gentamicin C_1, C_{1a} and C_2), levofloxacin, neomycin (main component neomycin B), tetracycline, tobramycin and trimethoprim were obtained from Sigma-Aldrich (St. Louis, MO, USA). Stock solutions were prepared in Milli-Q water or DMSO. Minimum inhibitory concentrations (MICs) were determined in cation-adjusted Mueller-Hinton broth (MHB2) using the broth microdilution method as recommended by the Clinical and Laboratory Standards Institute (CLSI) and by EUCAST guidelines (ISO 20776-1:2019). In short, serial dilutions of antibiotics were prepared in MHB2 in sterile 96-well plates and the bacterial suspensions were added. Growth inhibition was assessed after incubation for 18 h at

37 °C and the MIC was defined as the lowest concentration of the antibiotic causing complete inhibition of visible growth of the microorganism (CLSI, 2017)[3]. The validity of colistin MIC data was ensured by quality control testing with a susceptible *E. coli* strain (ATCC25922) and testing against the Col^R *E. coli* NCTC13846. One clinical isolate was additionally tested in M9 minimal medium (containing 1% glycerol, 2 mM $MgSO_4$ and 0.1 mM $CaCl_2$).

Large scale protein expression of ComG

Protein expression of ComG was started by transferring a freshly transformed *E. coli* Lemo21 (DE3) colony with the pET28b+-ComG vector into 100 mL LB-medium supplemented with 50 µg/mL kanamycin and 34 µg/mL chloramphenicol. The cultures were grown for 16 h at 37 °C and 200 rpm and subsequently used to inoculate an expression culture of LB-medium supplemented with the appropriate antibiotics at a ratio of 1:100. This culture was grown at 37 °C and 200 rpm until an OD_{600} of 1.0 was reached, at which point the temperature was decreased to 25 °C and the expression induced with 0.1 mM IPTG, after which the cultures were grown at the aforementioned temperatures for 16 h. Cultures were harvested by centrifugation at 5,000 rpm and 4 °C for 10 min, after which the respective cell pellet was collected and the supernatant discarded. Cell pellets were frozen at -80 °C until further use.

Selenomethionine expression of ComG

For seleno-methionine (SeMet) expression, a culture of LB-medium with the appropriate antibiotics was inoculated with a single, freshly transformed *E. coli* colony carrying the vector with the crystallized construct. For every liter of prepared medium for expression, 50 mL of LB medium were grown at 37 °C and 200 rpm for 16 h. The cultures were centrifuged at 2,800 rpm and 20 °C for 15 min and the cell pellet washed three times in M9 medium (8.5 g/L Na_2HPO_4, 3 g/L KH_2PO_4, 1 g/L NH_4Cl, 0.5 g/L NaCl) before inoculating M9 medium supplemented with glucose-free nutrient mix and 5% glycerol at a ratio of 1:20. The cultures were grown for 20 min at 37 °C at which point 40 mg/L L-selenomethionine was added to the culture. The cultures were then grown at 37 °C and 200 rpm until an OD_{600} of 0.6 was reached, where upon 100 mg/L each of L-lysine, L-phenylalanine and L-threonine and 50 mg/L each of L-isoleucine and L-valine were added. The cultures were incubated for another 20 min before the protein expression was induced by the addition of 1 mM IPTG. The temperature was reduced to 20 °C and the cells were grown for 24 h. The cells were harvested by centrifugation (5,000 rpm, 4 °C, 10 min).

Protein purification of ComG

ComG was inserted into pET28b+ with an N-terminal 6x His-tag using according primers. The cell pellet was resuspended in lysis buffer (150 mM NaCl, 20 mM imidazole, 25 mM Tris-HCl, pH 7.5), sonicated and centrifuged. The supernatant was loaded onto a gravity flow column containing Ni-NTA loaded sepharose, washed and incubated for 60 min with lysis buffer containing additional 5 mM ATP/$MgCl_2$ and subsequently eluted in one step (250 mM imidazole). 6x His-tagged fusion protein was passed through a Superdex 200 16/60 pg column in SEC buffer (150 mM NaCl, 25 mM Tris, pH 7.5), concentrated using a 10 kDa cutoff filter and stored at -80 °C in 10% glycerol. Protein purity was determined by SDS-PAGE. Protein concentration was determined spectrophotometrically upon determining the respective extinction coefficient from the amino acid sequence using the ProtParam webserver[4].

For crystallography, a ComG construct with an additionally cloned N-terminal TEV protease recognition site was used (see Cloning of *comG*).The cell pellets were resuspended in an optimized lysis buffer (500 mM NaCl, 20 mM Tris pH 8.0, 20 mM Imidazole, 3 mM β-mercaptoethanole). For every 25 g of wet cell mass, 100 mL of lysis buffer were added and supplemented with 2 cOmplete EDTA-free protease inhibitor tablets (Sigma-Aldrich) and 4 mg DNase (Sigma-Aldrich). Cell lysis was carried out via passage through a cell disruptor (30 kpsi, Constant Systems), and the cell debris was removed by centrifugation (19,000 rpm, 15 min, 4 °C). The supernatant was decanted, filtered through a 0.45 µm filter and applied to a His-Trap HP 5 mL column (GE Healthcare) pre-equilibrated in lysis buffer at a flow rate of 5 mL/min. The column was extensively washed with 150 mL lysis buffer and the target protein eluted using lysis buffer supplemented with 250 mM imidazole. The protein was passed over a desalting column (16/10 GE Healthcare) at a flow rate of 10 mL/min pre-equilibrated in desalting buffer (1 M NaCl, 20 mM Tris pH 8.0, 20 mM Imidazole, 1 mM DTT). To remove the His-tag, the desalted protein was incubated with TEV protease for 14 h and 4 °C at a 1:10 mass ratio of TEV:target protein. Subsequent passage of the solution over a 5 mL His-Trap HP column allowed for a separation of the digested target protein and the 6x His-tag. The protein was then passed over a Superdex 200 16/600 size exclusion column (GE Healthcare) at a flow rate of 1 mL/min pre-equilibrated in gel filtration buffer (1 M NaCl, 10 mM HEPES, 5% glycerol, 0.5 mM TCEP, pH 7.4). The resulting peak was collected and concentrated to the desired concentration using a 30 kDa cutoff filter (Thermo Scientific). The protein concentration was determined using photometric analysis (Nanodrop 2000, Thermo Scientific) and subsequently analyzed by SDS-PAGE. Protein identity was additionally confirmed by intact protein mass spectrometry.

Crystallization of ComG

Initial crystallization trials of apo ComG were set up using a protein concentration of 200 µM at 4 °C. Crystals were observed after 2 days in 200 mM $MgCl_2$, 100 mM Tris pH 8.5 and 20% PEG 8000. Selenomethionine-labelled ComG could be crystallized in the same condition and optimized by a variation of the aforementioned chemicals and pH screening in the well solution. Single crystals were cryo-protected by supplementing the crystallization solution with 32% glycerol, mounted into cryoloops (Hampton Research) and flash frozen in liquid nitrogen. SeMet-ComG crystals gave better data than native ComG crystals. A high-redundancy dataset from SeMet-ComG crystals at the selenium K-edge was collected at the Swiss Light Source (SLS) Beamline X10SA.

For ComG complex crystallization, a fresh batch of protein was purified as described above, with the exception of the gel filtration buffer being 1 M NaCl, 10 mM Tris, 0,5 mM TCEP, 10% glycerol, pH 8.0. Co-crystallization was carried out by incubating 200 µM of ComG with 2 mM corramycin and 5 mM AMPPCP/$MgCl_2$ for 16 h on ice before setting up crystallization trials. Single $ComG^C$ crystals of 100 – 200 µm were observed in multiple screening conditions. They were cryoprotected by supplementing the crystallization solution with 32% glycerol, mounted into cryoloops (Hampton Research) and flash frozen in liquid nitrogen. A complete dataset was collected from a crystal grown in condition PEGS D5 (0.1 M Sodium HEPES pH 7.5, 20% PEG 10000) at a wavelength of 0.97 Å at beamline X06SA located at the Swiss Light Source.

Data processing, structure determination, refinement and structural analysis of ComG

All data were processed using XDS[5] and POINTLESS[6], AIMLESS[7] and Ctruncate implemented in ccp4[8]. The apo structure of ComG was determined using Phenix.AutoSol[9] (Se-SAD) followed by several rounds of manual rebuilding in COOT[10] and refinement in Phenix.refine[11]. The structure of $ComG^C$ was determined by molecular replacement (*phenix.phaser*[12]) using the apo ComG structure as a search model. The $ComG^C$ structure also underwent several rounds of manual rebuilding in COOT[10] and refinement in Phenix.refine[11]. Final PDB coordinates were analyzed using MolProbity[13], used for detection of macromolecular assemblies with the PISA server[14] as well as for structural homology analysis on the DALI server[15]. All structural images portrayed were rendered in PyMOL (The PyMOL Molecular Graphics System Version 1.8.6.0, Schrödinger, LLC).

ComG *in vitro* assay

10 µM ComG, 1 mM drug (corramycin/capreomycin) and 1 mM ATP (or GTP) were incubated in reaction buffer (150 mM NaCl, 10 mM $MgCl_2$, 25 mM Tris-HCl, pH 7.5) in 30 µL final volume

at 22 °C for 2 h. Drug incubated with ComG alone (without ATP/GTP, Figure S3) and drug incubated with solely ATP/GTP (without ComG) served as negative controls. Samples were diluted 1:100 with Milli-Q water and protein was precipitated by addition of an equal volume of MeOH. Precipitated protein was removed by centrifugation (15,000 rpm, 10 min 4 °C) and the supernatant was subjected to LC-MS analysis. For purification of the phosphorylated corramycin (Cor-P) the assay was scaled to 50 mL volume and the incubation time was increased to 16 h, the supernatant was lyophilized and subjected to semi-preparative HPLC.

Kinetic parameters were determined by incubating 15 µM ComG, 4 mM ATP and various corramycin concentrations (10, 50, 100, 200 and 300 µM) for different time-spans (0, 1, 5, 10, 30, 45 and 60 min) at 30 °C in a total volume of 15 µL in a 96-well plate. The reaction was stopped by adding trichloroacetic acid to a final concentration of 5% (*w/v*). Precipitated protein was removed by centrifugation (15,000 rpm, 10 min, 4 °C) and the supernatant was subjected to LC-MS analysis. Michaelis-Menten kinetic parameters were determined by quantifying the initial velocity at the different corramycin concentrations using EIC peak areas of singly and doubly protonated Cor-P. All experiments were carried out in triplicates.

Sample measurements were performed using a Dionex Ultimate 3000 RSLC system (Thermo) coupled to a maXis 4G q-TOF mass spectrometer (Bruker Daltonics). 1 µL of the sample was loaded on the BEH C18 column (100 × 2.1 mm, 1.7 µm d_p) (Waters) and separation was achieved by gradient elution (eluent A: H_2O + 0.1% FA, eluent B: ACN + 0.1% FA) at a flow rate of 600 µL/min and 45 °C. The analysis was initiated by 0.5 min isocratic flow with 5% B, followed by a linear increase to 95% B in 18 min to end with 2 min holding 95% B before equilibration with 5% B. LC flow was split to achieve a flow rate of 75 µL/min entering the Apollo II ESI source (Bruker Daltonics). The split was set up using fused silica capillaries of 75 and 100 µm I.D. and a low dead volume tee junction (Upchurch). Prior to sample elution, each analysis started with a calibrant peak of basic sodium formate solution for internal calibration. Range of mass spectra acquired was set from 150 – 2,500 *m/z* at 2 Hz scan rate in centroid mode. Capillary voltage was set to 4,000 V with an end plate offset of 500 V for measurements in positive ionization mode. Dry gas flow rate was set to 5 L/min at 200 °C dry temperature. Full scan spectra were acquired at 2 Hz like described above, followed by MS^2 spectra acquisition with a scan speed of 0.68 Hz. CID energy varies linearly from 35, 45, to 60 eV for precursor *m/z* from 500, 1000, to 2000 *m/z* respectively. The ion cooler was set to ramp collision energy (80 − 120% of the set value) and ion cooler RF from 700 to 1000 Vpp for every MS/MS scan. Precursor intensity threshold was set to 5000 cts.

Purification, resistance validation and structure determination of Cor-P

Freeze-dried 50-mL *in vitro* assay was resuspended in methanol and purified using semi-preparative HPLC. Purification was conducted using a Dionex HPLC system (Famos autosampler, P680 pump, TCC100 thermostat, and PDA100 detector) equipped with a Phenomenex Luna C18, 250 x 10 mm, 80 µm dp column. Separation was achieved by a linear gradient using (A) H_2O + 0.1% FA and (B) ACN + 0.1% FA at a flow rate of 5 mL/min and 30 °C. The gradient started at 10% B with a 3 min hold and increased to 70% B in 15 min (4% B/min). Subsequently, B was increased to 95% in 1 min with a 1 min hold. A UV spectrum was acquired at 200 – 600 nm. The sample was injected by µL-pick-up technology with a water/methanol (50:50 *v/v*) mixture as supporting solvent. A maximum of 50 µL (6 µg / µL) of the sample was injected before manual fraction collection. Purity of fractions was tested using the LC-MS method described above. NMR spectra were recorded on a 700 MHz Avance III (Ascend) spectrometer by Bruker BioSpin GmbH, equipped with a 5 mm TXI cryoprobe, at 298 K. Chemical shift values of ^{1}H- and ^{13}C-NMR spectra are reported in ppm relative to the residual solvent signal given as an internal standard. ^{13}C-signals were assigned via 2D-CH and CCH correlations (HSQC and HMBC) (Figure S7A). The inhibitory concentration of Cor-P was determined using an adapted broth microdilution method. An overnight culture of *E. coli* BL21 (DE3) was diluted 1:100 and grown until mid-logarithmic phase was reached. Optical density (OD_{600}) of the bacterial culture was adjusted to a cell count of ~2x10^6 CFU/mL and equal volume of this bacterial suspension was dispensed in a 96-well plate containing increasing concentrations of the native as well as the phosphorylated corramycin (in Luria-Bertani broth). The plate was incubated for 18 h (30 °C, 300 rpm) and the inhibitory concentration was defined as the lowest concentration causing complete inhibition of visible growth.

Molecular dynamics (MD) simulations

The system, comprising the protein ComG in complex with ATP, Mg^{2+} and the ligand (corramycin), in a cubic water box with the size of about 9 nm, was constructed using the CHARMM-GUI web server[16,17]. The ComG^c structure was used as the initial coordinates of the protein with the corramycin ligand. The initial position of the ATP and the Mg^{2+} center was determined from the binding of this cofactor to the catalytic center of the choline kinase from Cryptosporidium parvum Iowa II (PDB ID 3mes) by aligning the protomers with PyMOL. Cl^- ions were added to neutralize the protein charge and K^+ and Cl^- ions were added up to a final concentration of 0.15 M KCl. The system was described using the CHARMM36m Force Field[18] and the ligands were parametrized using the CHARMM General Force Field[19]. The TIP3P model was used to describe the water molecules[20]. The simulations were conducted using

GROMACS 2019 and the results analyzed with GROMACS tools[21]. Simulations were visually analyzed with the VMD software (version 1.9.4.)[22].

The system was first minimized using the steepest descendent algorithm until the maximum force of 10.0 kJ mol^{-1}. Equilibration comprised a first 125 ps run with a canonical ensemble and random initial velocity generation, using an integration step of 1 fs followed by 10 subsequent productions of 1 ns, using an isothermal-isobaric ensemble, without position restraints for the protein and ligands. Final production consisted in 100 ns simulation using the isothermal-isobaric ensemble. The protocol (minimization + equilibration + discussion) was repeated 4 times with different initial velocities to ensure that the dynamics of the catalytic center is reproducible. Temperature was set to 300 K using the Nose-Hoover thermostat[23,24] with a coupling constant of 1.0 ps. Pressure was set to 1.0 bar using the Parrinello-Rahman barostat[25] with a coupling constant of 5 ps. Long-range electrostatic interactions were treated by the particle-mesh Ewald (PME) method[26,27] with a real space cut-off of 1.2 nm. The van der Waals interactions were treated with a cut-off of 1.2 nm. H-Bonds were constrained using the LINCS algorithm[28] and the SETTLE algorithm[29] was used for the water molecules.

Bioinformatics analysis

Sequence similarity searches were conducted with BLAST[30] using blastP and tblastn programs. For the phylogenetic search an E-value cut-off of 10^{-5} was used, for all other searches the default cut-off of 0.05 was used. If needed, the corresponding nucleotide sequences were downloaded and proteins were extracted by Prokka[31]. The multiple sequence alignment was built with MAFFT[32]. The phylogenetic tree was built with FastTree[33]. Corramycin-binding residues were identified as all residues lying closer than 5 Å from the bound corramycin. The corresponding positions were extracted from the multiple sequence alignment with a custom Perl script, and the sequence logo was built with Weblogo 3[34]. To investigate ComG specificity to nucleotide, we considered all three-dimensional structure containing a nucleotide-like cofactor from the Pfam Phosphotransferase enzyme family (PF01636, Table S6). For atoms of the adenine and guanine bases (N6, C6, N1, C2, N3, C4, C5, N7, C8, N9 and O6, C6, N1, C2, N2, N3, C4, C5, N7, C8, N9, respectively), spatially closest amino acid residues in the protein were found, and based on these residues, a sequence logo was constructed with Weblogo 3[34].

Supplementary Figures

```
CLUSTAL O(1.2.4) multiple sequence alignment


ComG                      ---MGNNSRASNPVPLEDQIGALLGEHPRQIVPLHAGRRAQVLRCHFQDGHSVIVKSFTE    57
Myxococcus_sp_CA040A      MKKADTQRKVVRLGFVADQVHDLMGERPRRLETLQQGLRARVLRCHLRGGRSVIVKASTE    60
Myxococcus_llanfairpw     MKKADTQRKVVRLGFVADQVHDLMGERPRRLETLQQGLRARVLRCHLRGGRSVIVKASTE    60
                              ..: :. .   : **:  *:**:**::  *: * **:*****::.*:*****: **

ComG                      VAETTRGEWDALRFLAAHVPAIAPRPLARSKDRRLVAMEDLRGETLARLLERESEAGARR    117
Myxococcus_sp_CA040A      VTQTGRREWEALRFLSARAPSLAPRLLGRSKDRRLVIMEDLKGETLARLLERESESGARK    120
Myxococcus_llanfairpw     VTRTGRREWEALRFLSARAPSLAPRLLGRSKDRRLVIMEDLKGETLARLLERESESGARK    120
                          *:.* * **:*****:*:.*::*** *.******** ****:*************:***:

ComG                      PLVRIADALGHLHGAQAPRVDGLPRALRDEYRKQADECVALRGKVRALLGRAGVEPTPGF    177
Myxococcus_sp_CA040A      PLVSIAERLGHFHGQQVTRLDSLPRSLRGEYLQQAKDCVSLQGRVLGLLERAGVKPSPGF    180
Myxococcus_llanfairpw     PLVSIAERLGHFHGQQVTRLDALPRSLRGEYLQQAKDCVSLQGRVLGLLERAGVKPSPGF    180
                          *** **: ***:** *. *:*.***:**.** :**.:**:*:*:* .** ****:*:***

ComG                      DGAWLELVERMGSPGPFLTFTHGDLAPSNVLLTDDGPRLLDFEYTGARSALYDVMFWEAV    237
Myxococcus_sp_CA040A      DGAWQELVERMGSPGAFLTVTHGDLAPSNVLLTPAGPRLLDFEYTGARSALYDVMFWEFV    240
Myxococcus_llanfairpw     DGAWQELVERMGSPGAFLTVTHGDLAPSNVLLTPAGPRLLDFEYTGARSALYDVMFWEFV    240
                          **** ********** ***.*************  *********************** *

ComG                      VPFPRSLARPMTQAYRRALASHLPAARDDARFRRELLTLKTHRFFWWLTFRLDEALAGGD    297
Myxococcus_sp_CA040A      VPFPRTLARAMTRAYRTALGAGLPEARDEARFRQELATLKTHRFYWWLTFRLAEALAGAD    300
Myxococcus_llanfairpw     VPFPRTLARAMTRAYRTALGAVLPEARDEARFRQELATLKTHRFYWWLTFRLAEALAGAD    300
                          *****:*** **:*** **.: ** ***:****:** *******:******* *****.*

ComG                      AHWVPGWRLRPAYLFYLQNYVSTARRLGARGPLLKTAQALSSRLRRGWKERAGYPDHFLG    357
Myxococcus_sp_CA040A      AHWVPGWRLRPAYLFYLQNFLTTSRSLGVEGPLTRTARALATRLREQWVDRAGYPDHFLP    360
Myxococcus_llanfairpw     AHWVPGWRLRPAYLFYLQNFLTTSRSLGVEGPLTRTARALATRLREQWVDRAGYPDHFLP    360
                          *******************:::*:* **..*** :**:**::***. * :*********

ComG                      KLKPPGP--    364
Myxococcus_sp_CA040A      APSLQGRRG    369
Myxococcus_llanfairpw     APSLQGRRG    369
```

Figure S1. Sequence alignment of ComG from *Corallococcus coralloides* with similar genes from two different *Myxococcus* species (sequences from *Myxococcus* species are identical). The sequence identity was determined to be 72.5%. The alignment was generated with Clustal Omega[35].

A

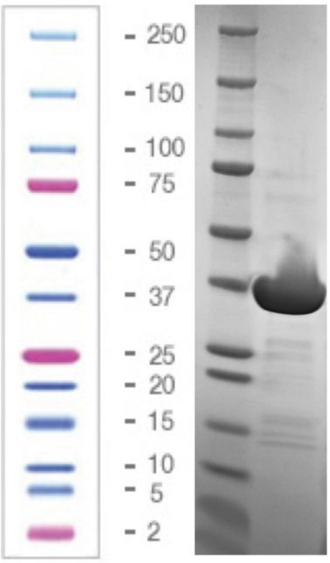

B

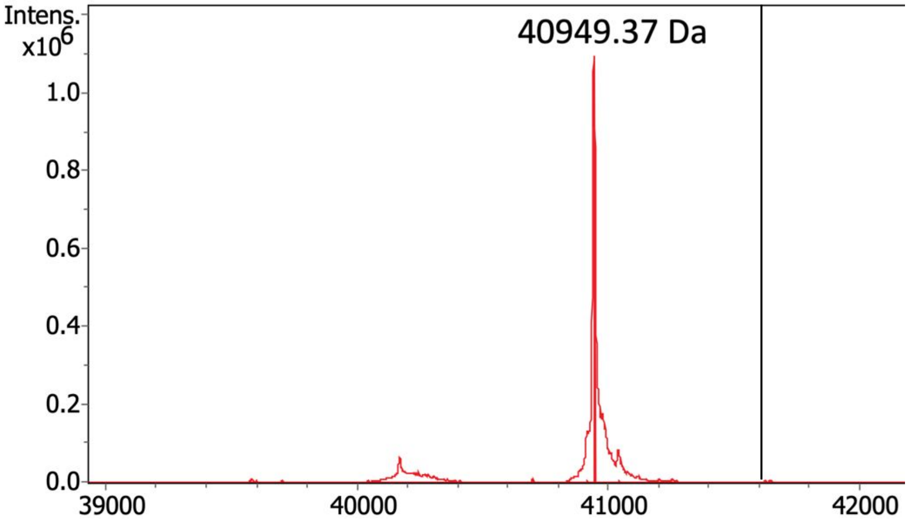

Figure S2. **A** SDS-PAGE analysis of a purified ComG sample. Precision plus dual xtra prestained ladder was used as a protein standard. **B** HR-LCMS analysis of a purified ComG sample. Maximum entropy deconvolution was used to determine the mass of the protein (calculated mass: 40,949.97 Da; observed mass: 40,949.37 Da).

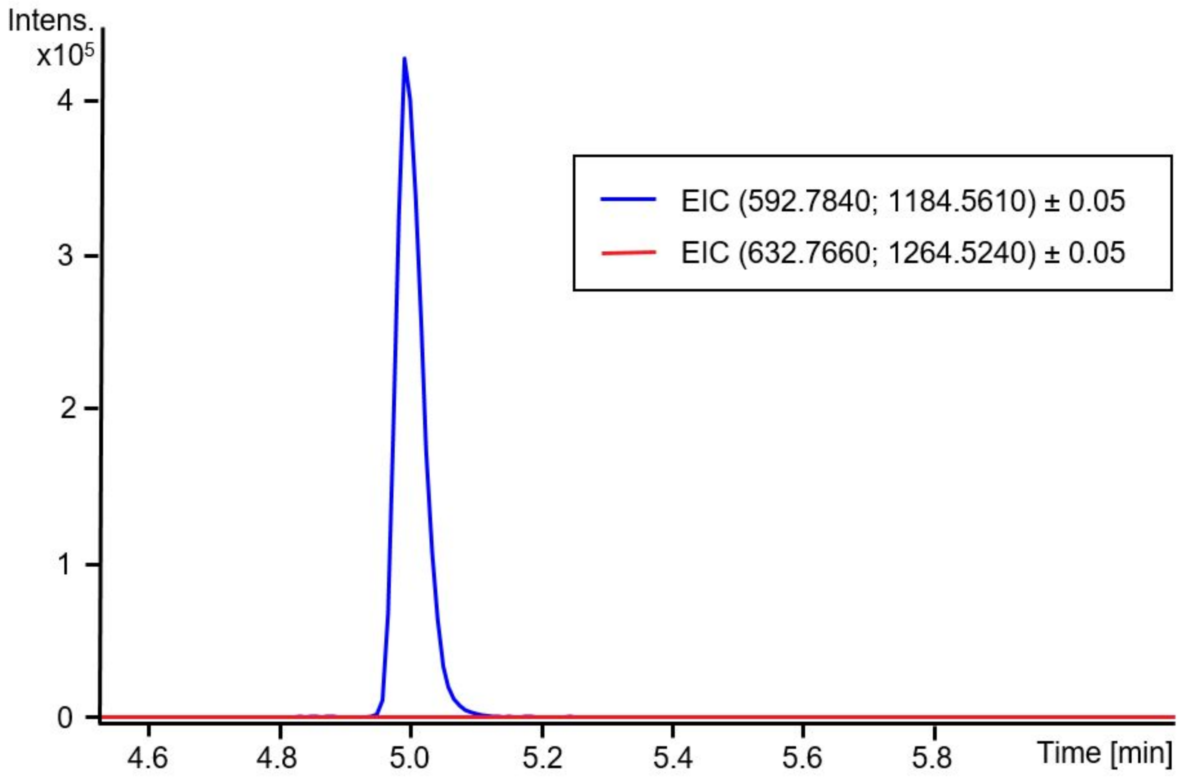

Figure S3. Extracted ion chromatogram (EIC) of an *in vitro* reaction of corramycin with ComG (without ATP). ComG does not phosphorylate corramycin in the absence of ATP.

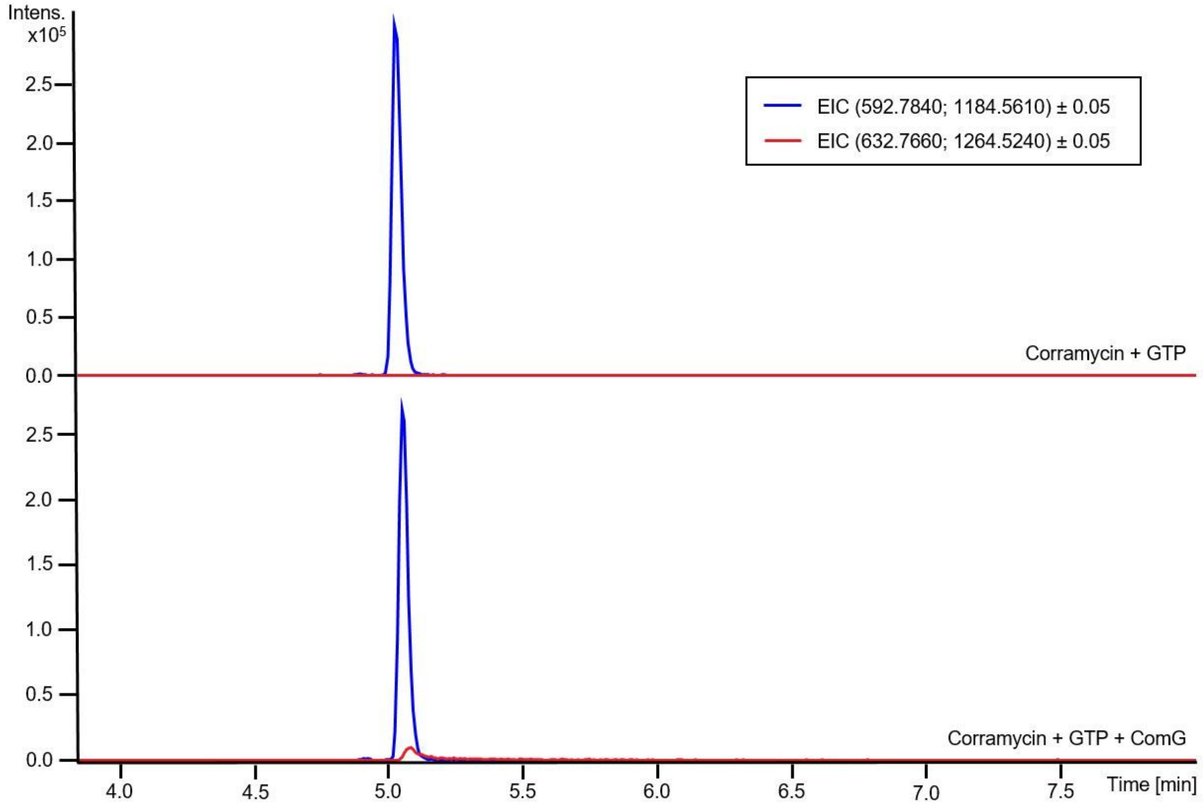

Figure S4. Extracted ion chromatograms (EICs) of an *in vitro* reaction of corramycin with ComG and GTP/$MgCl_2$. The majority of corramycin remains unphosphorylated when GTP was used as cofactor, indicating that ATP is the preferred phosphate donor of ComG.

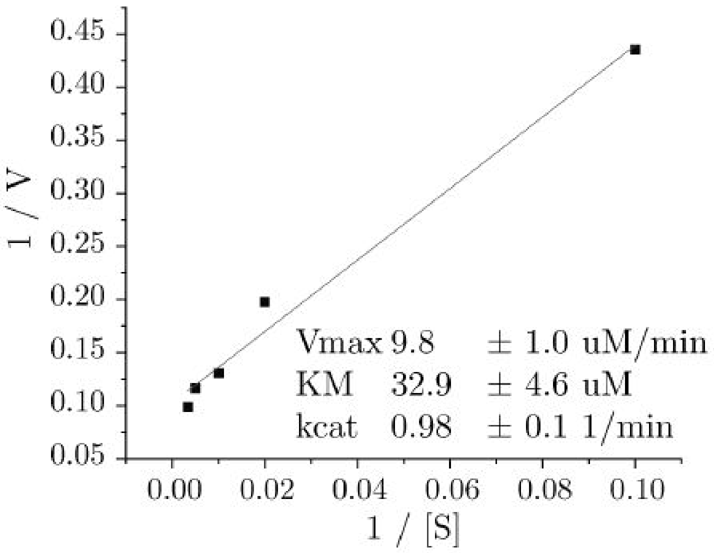

Figure S5. Lineweaver-Burk plot of the ComG reaction with a series of corramycin concentrations and ATP as phosphate donor. Michaelis-Menten kinetics were determined by quantification of EIC signal areas of Cor-P. Linear fit was used to calculate kinetic parameters (mean ± SD, n = 3).

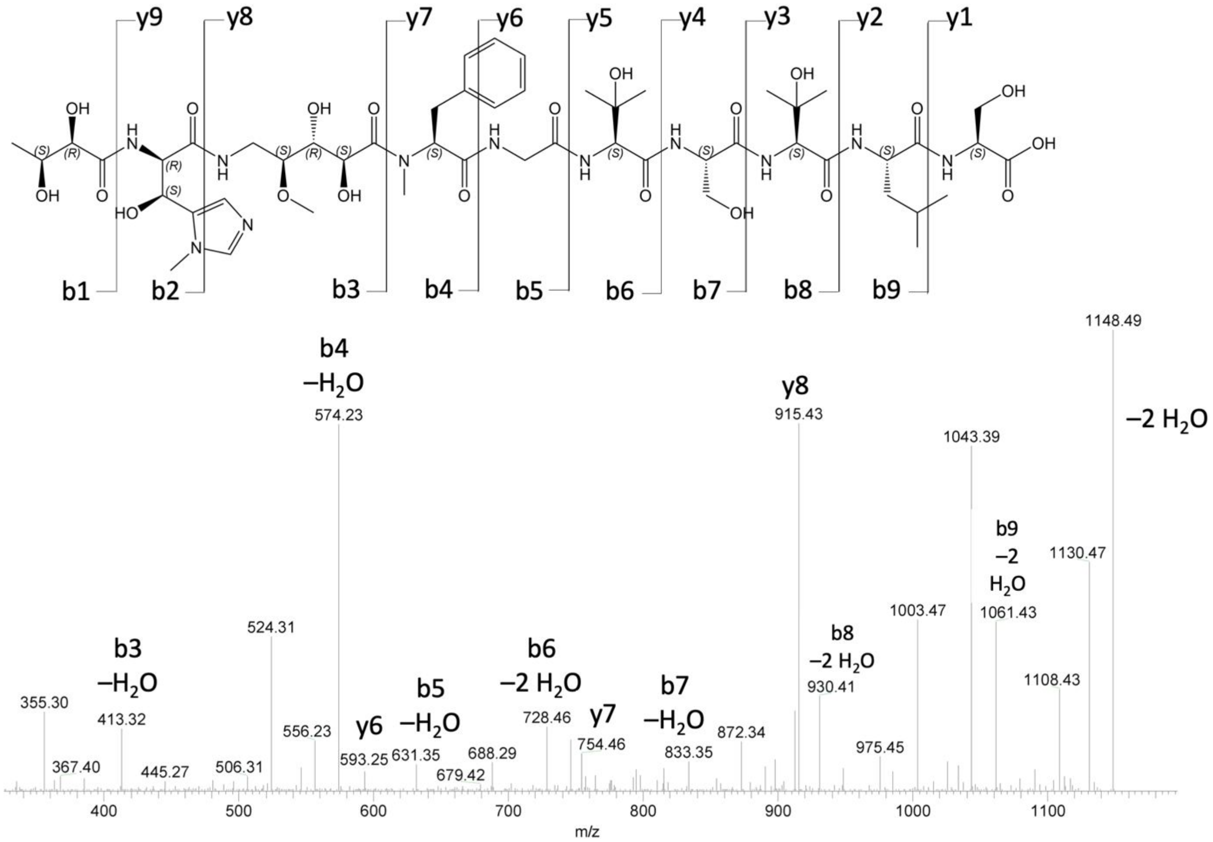

Figure S6. Tandem MS/MS analysis of corramycin. Characteristic peptide fragmentation and resulting y- and b-ions are shown.

A

Atom	Corr	P-Corr	Δ
2	68.3	68.2	-0.1
	4.51	4.81	0.3
3	70.2	73.4	3.20
	4.03	4.38	0.35
4	78.4	77	-1.4
	3.57	3.7	0.13
5	38.2	38.1	-0.1
	3.57	3.51	-0.06
		3.7	0.13

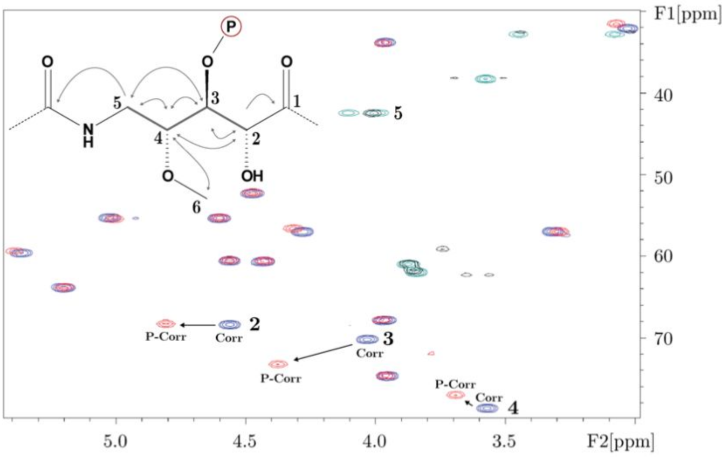

B

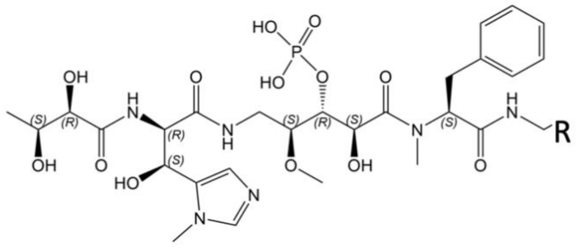

P-Corramycin

Chemical Formula: $C_{51}H_{82}N_{11}O_{24}P$
Exact Mass: 1263,53

Figure S7. **A** NMR analysis of the phosphorylated corramycin (Cor-P). Superimposed HMBC-spectra of corramycin (blue/dark green) and Cor-P (red/green) in combination with the calculated ppm shifts of the β-alanine PKS moiety locate the phosphate group at the C_3-hydroxyl-group position. **B** Determined structure of Cor-P.

10	20	30	40	50	60
GGMGNNSRAS	NPVPLEDQIG	ALLGEHPRQI	VPLHAGRRAQ	VLRCHFQDGH	SVIVKSFTEV
70	80	90	100	110	120
AETTRGEWDA	LRFLAAHVPA	IAPRPLARSK	DRRLVAMEDL	RGETLARLLE	RESEAGARRP
130	140	150	160	170	180
LVRIADALGH	LHGAQAPRVD	GLPRALRDEY	RKQADECVAL	RGKVRALLGR	AGVEPTPGFD
190	200	210	220	230	240
GAWLELVERM	GSPGPFLTFT	HGDLAPSNVL	LTDDGPRLLD	FEYTGARSAL	YDVMFWEAVV
250	260	270	280	290	300
PFPRSLARPM	TQAYRRALAS	HLPAARDDAR	FRRELLTLKT	HRFFWWLTFR	LDEALAGGDA
310	320	330	340	350	360
HWVPGWRLRP	AYLFYLQNYV	STARRLGARG	PLLKTAQALS	SRLRRGWKER	AGYPDHFLGK

LKPPGP

Figure S8. Primary amino acid sequence of ComG. The characteristic hinge region residues are highlighted in yellow, while the Mg^{2+}-coordinating residues are highlighted in red. The “FE” of the characteristic kinase DFG motif mutated to DFE in ComG is highlighted in green.

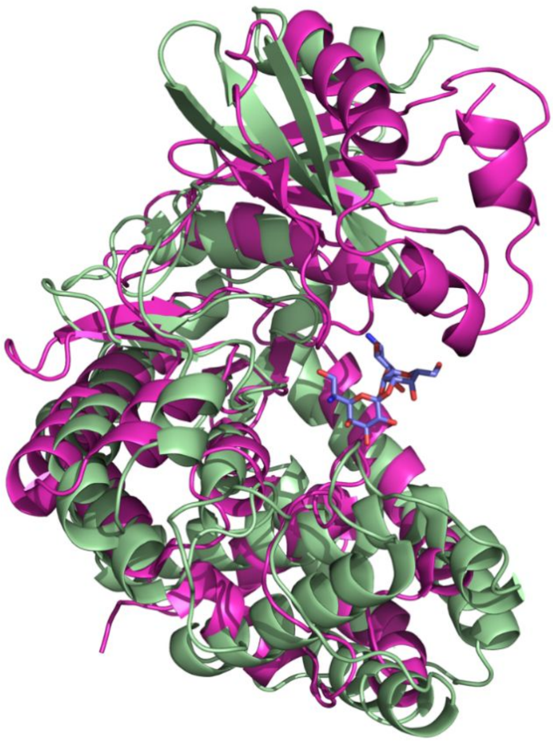

Figure S9. Superposition of the cartoon representations of apo ComG (palegreen) with PDB ID 6IY9 (magenta). Hygromycin B substrate of 6IY9 is shown as blue sticks. The C_{α} RMSD was calculated to be 3.73 Å over 190 atoms.

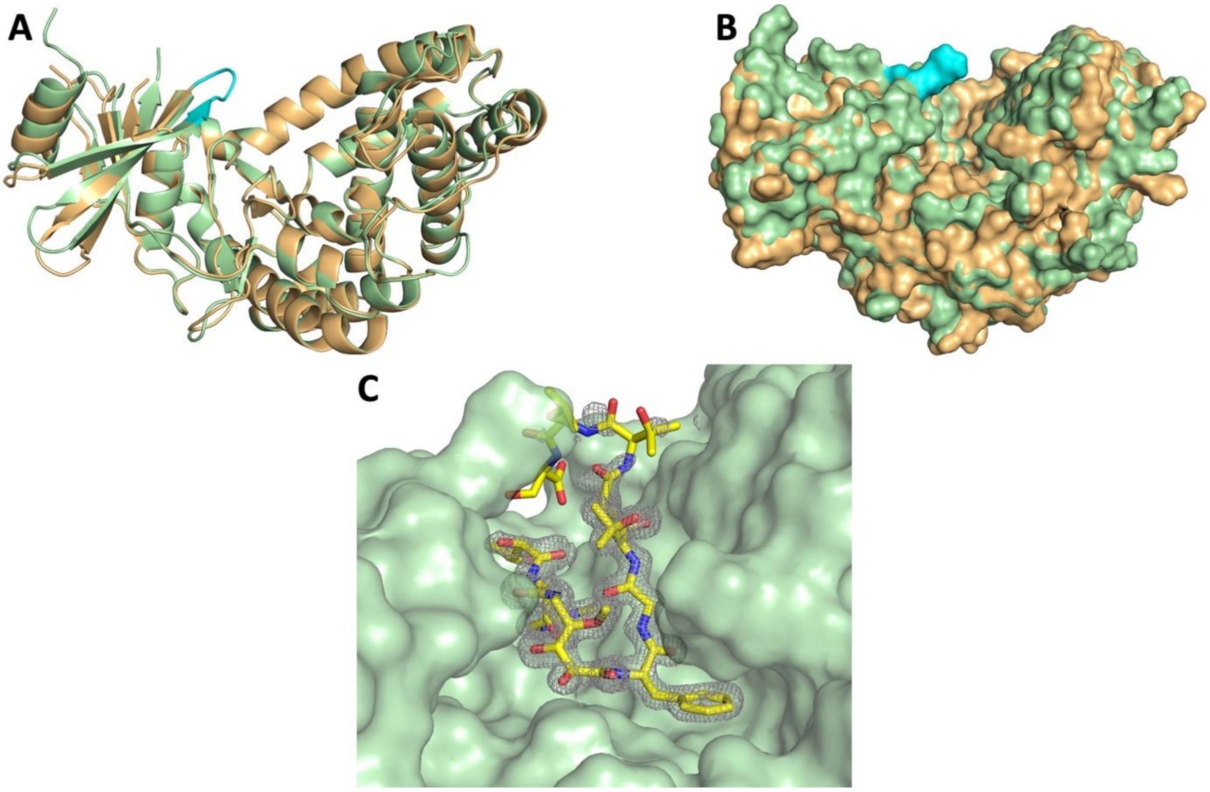

Figure S10. Crystal structure of the ComG-corramycin complex. **A** Superposition of apo ComG (sand) with ComG^C (green). The loop ordered in the complex structure is shown in cyan. **B** Surface representation of **A** (same color scheme). Arg38 (large, cyan protrusion) appears to be the main contributor to loop stabilization. **C** The difference electron density map of corramycin ($F_O - F_C$) was contoured at 3σ with phases calculated from a model that was refined in the absence of corramycin and is shown as a grey isomesh.

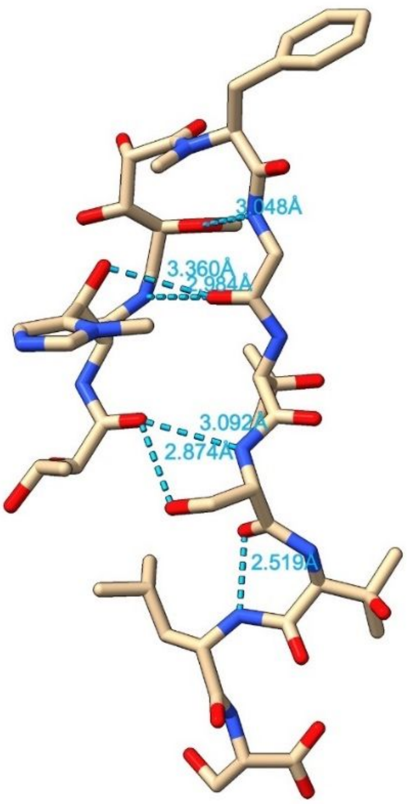

Figure S11. Intramolecular stabilization of corramycin. The V-shaped orientation of corramycin mimics an antiparallel β-sheet with six intramolecular hydrogen bonds (cyan dashes). Distances are given in Å.

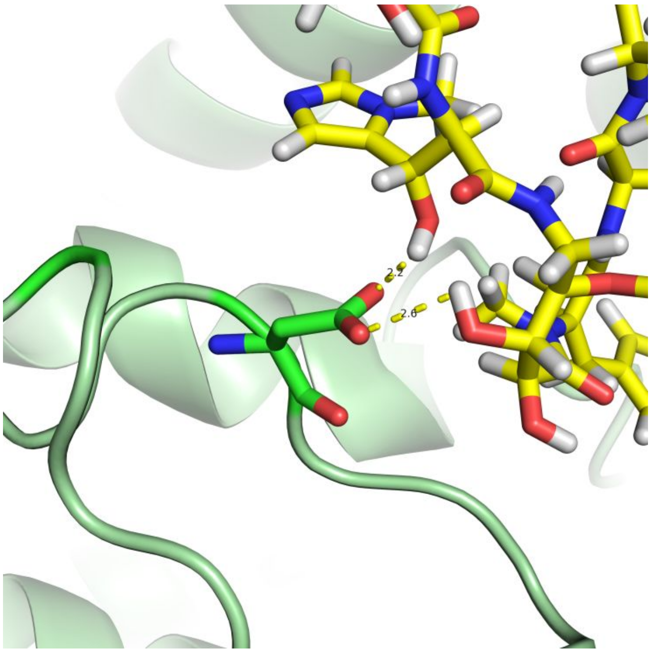

Figure S12. Coordination of the PKS-derived part of corramycin. The formation of two hydrogen bonds between Asp203 and hydroxy groups of corramycin coordinates both the γ-N-methyl-β-OH-histidine and 5-Amino-2,3-dihydroxy-4-methoxy-pentane moieties (distances 2.2 and 2.6 Å, respectively).

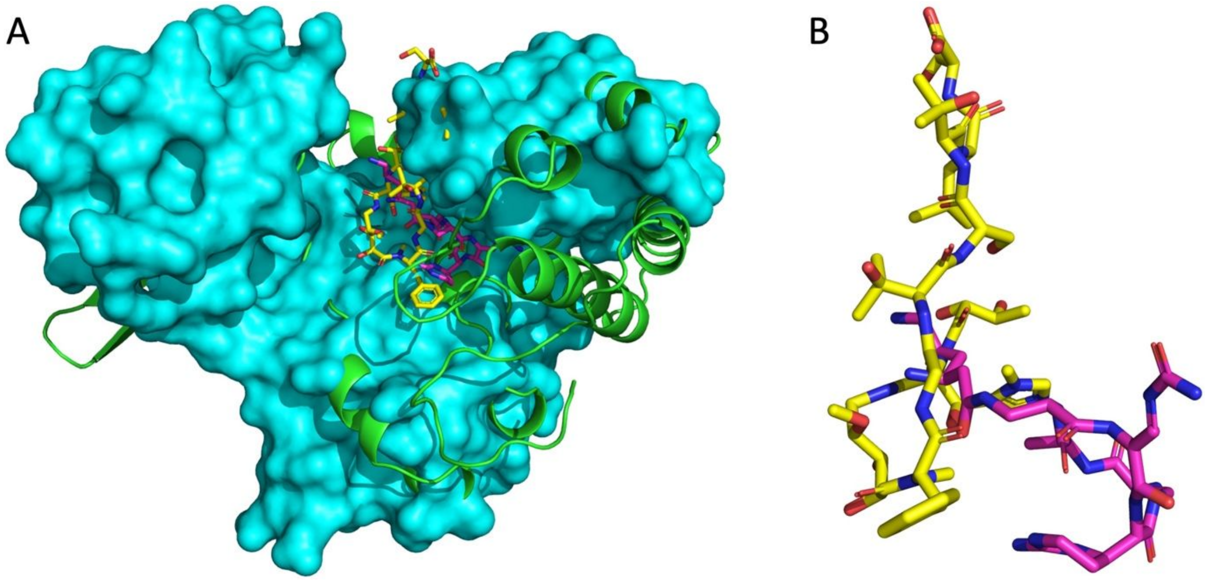

Figure S13. Structural comparison between the corramycin and capreomycin kinases. **A** Cph is shown as a surface representation in cyan, while ComG is shown as a green cartoon. The natural products corramycin and capreomycin are shown as yellow and magenta sticks, respectively. The Cα RMSD was determined to be 6.22 Å over 214 atoms. **B** Close-up of the superposition of corramycin and capreomycin as a result of the structural alignment shown in **A** with the same coloring. The compounds barely overlap, which emphasizes the differences in binding to their respective kinases.

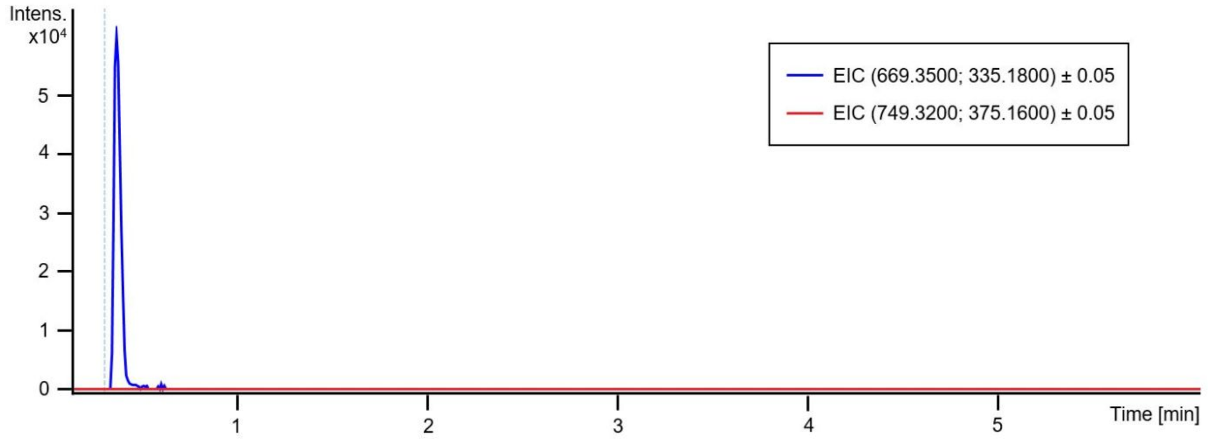

Figure S14. Extracted ion chromatogram (EIC) of an *in vitro* reaction of capreomycin with ComG and ATP/$MgCl_2$. Capreomycin is not a substrate for ComG, therefore no conversion is observed.

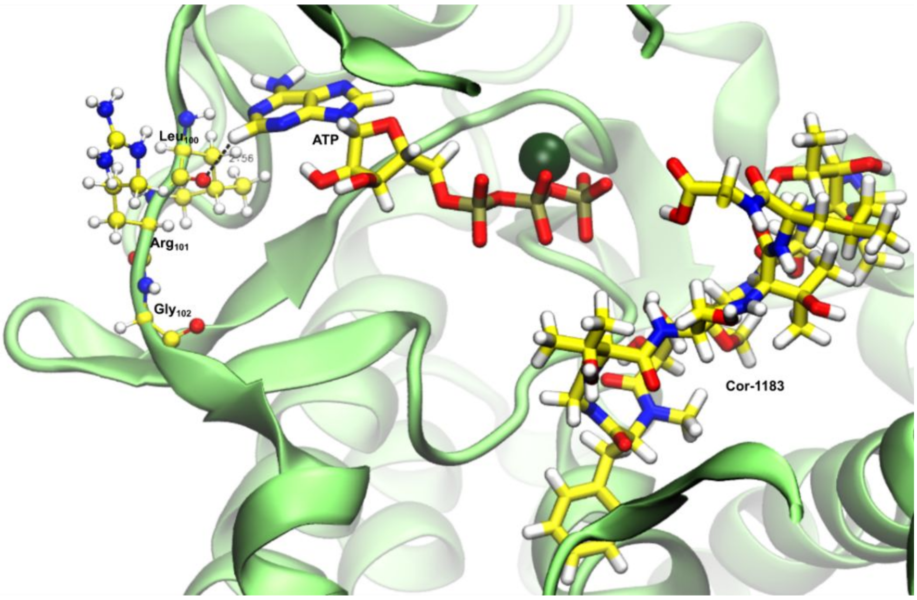

Figure S15. Modeled coordination of ATP with the hinge region of ComG. A hydrogen bond is formed between the oxygen of the backbone of Leu100 and the hydrogen from the adenine of ATP (distance ranges from 2 to 4 Å, along the simulated time).

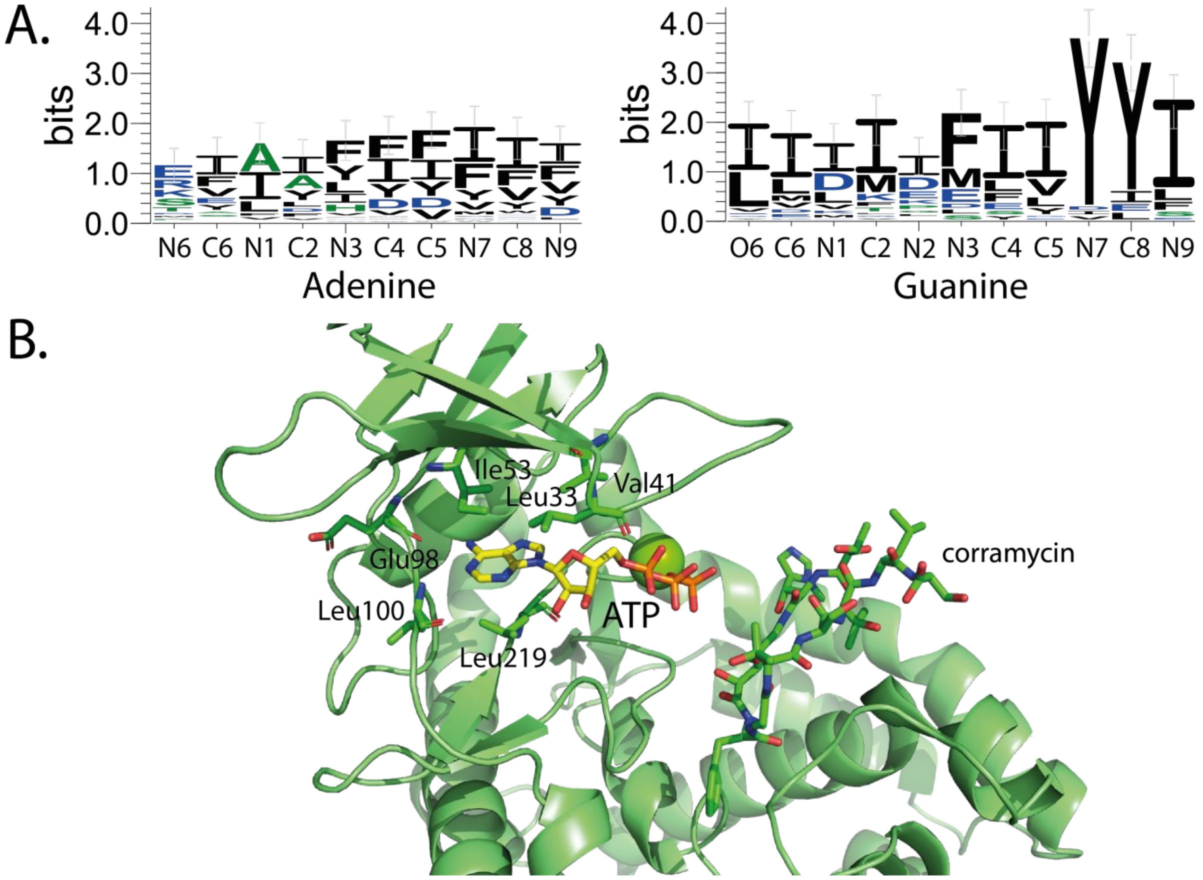

Figure S16. Structural basis of cofactor specificity of ComG. **A** Sequence logos for nucleobase-binding residues in the phosphotransferase enzyme family APH (Pfam family PF01636, Table S6). Note that same amino acid can be the closest residue for several atoms, then it appears in multiple columns of the logo. **B** Corresponding amino acid residues in the ComG structure (production 2, 80 ns).

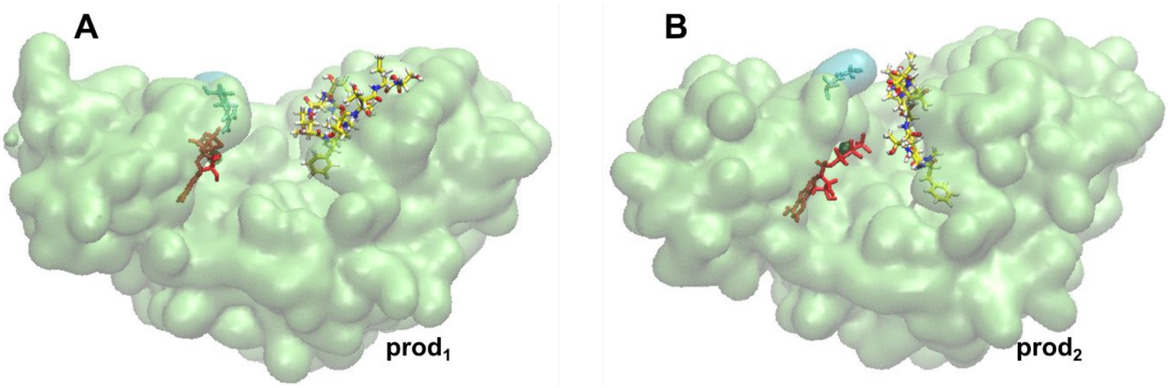

Figure S17. Representation of the position of the protein ComG, ATP and corramycin after 90 ns of simulation in production 1 (**A**) and production 2 (**B**). Protein surface is represented in pale green, corramycin is shown with yellow sticks and ATP with red sticks. The side chain of Arg37 is colored in cyan.

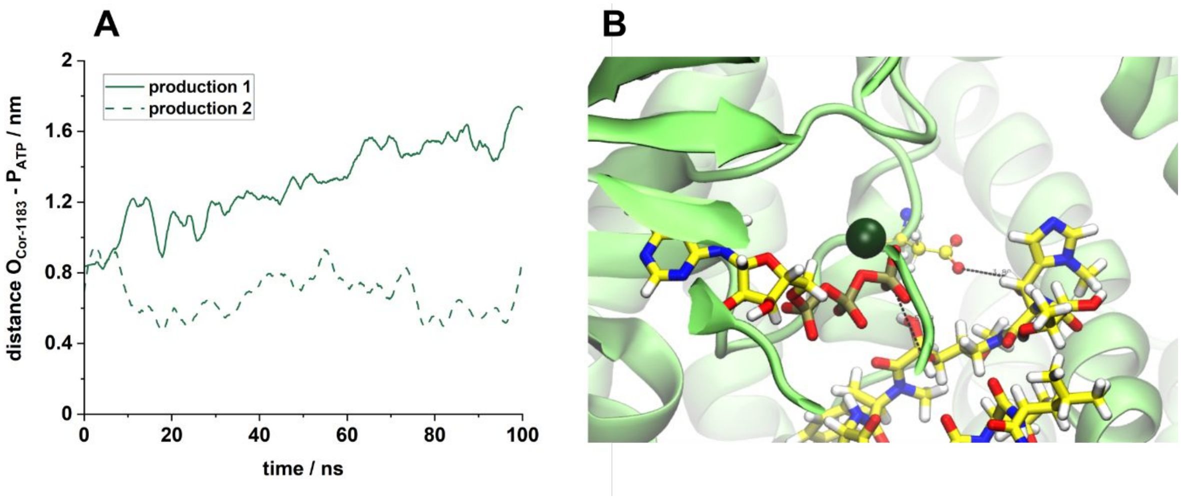

Figure S18. Modeled interaction between corramycin and ATP. **A** Distance between the oxygen of the C_3-hydroxyl group derived from the carboxy group of β-alanine from corramycin and phosphorous from the terminal phosphate from ATP. Results from production 1 are shown with solid lines and production 2 with dashed lines. Interaction between these two groups was only observed in one of the simulations (production 2). These results indicate that the orientation of the N-terminal part of corramycin is important for its interaction with ATP, as depicted in the comparison between the position of corramycin after 90 ns of simulation in production 1 and 2 (Figure S17). **B** Frame from production 2, representing the interactions between corramycin, the phosphate group from ATP and Asp203. Corramycin and ATP are represented with sticks, Asp203 with ball and sticks, and Mg^{2+} is shown in dark green. Carbon atoms are colored in yellow, nitrogen in blue, oxygen in red, phosphorous in gold and hydrogens in white. Protein backbone is shown in pale green.

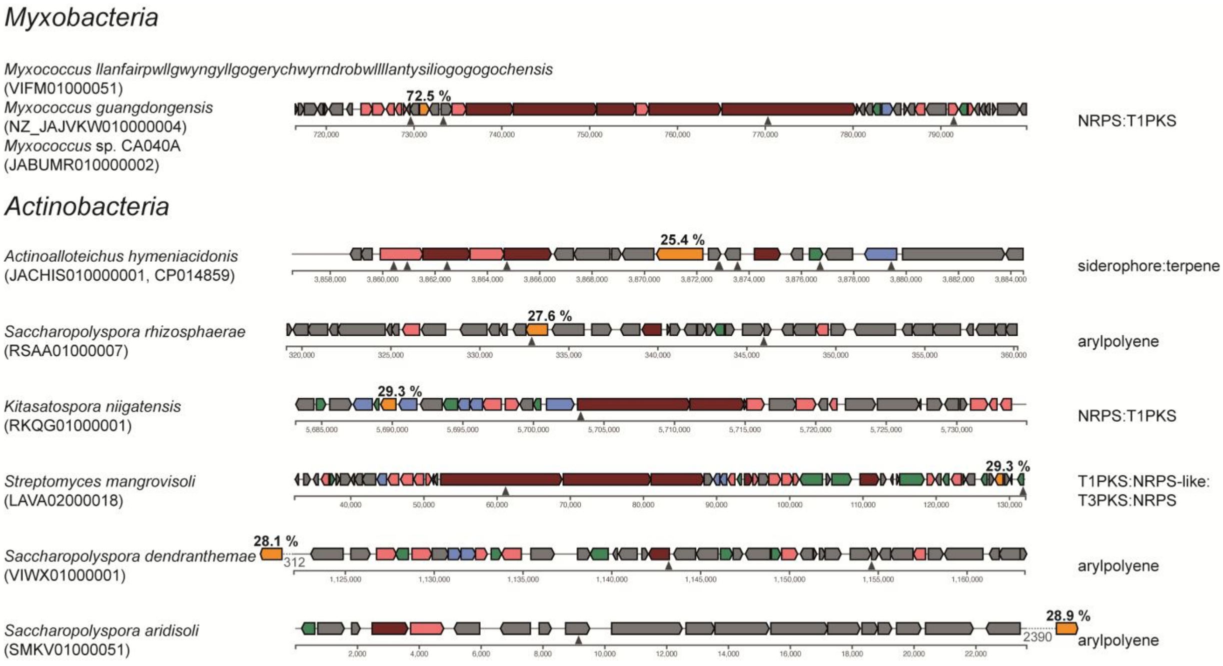

Figure S19. BGCs containing ComG homologues. ComG homologues are shown in orange, sequence identity to the *C. coralloides* protein sequence is displayed above the corresponding gene. In cases where the ComG lies outside a BGC, the region between it and the corresponding BGC is depicted as a dotted line, and the distance to the BGC border is shown below in bp. The BGCs were depicted using the antiSMASH website[36]. AntiSMASH annotations of BGCs are shown on the right.

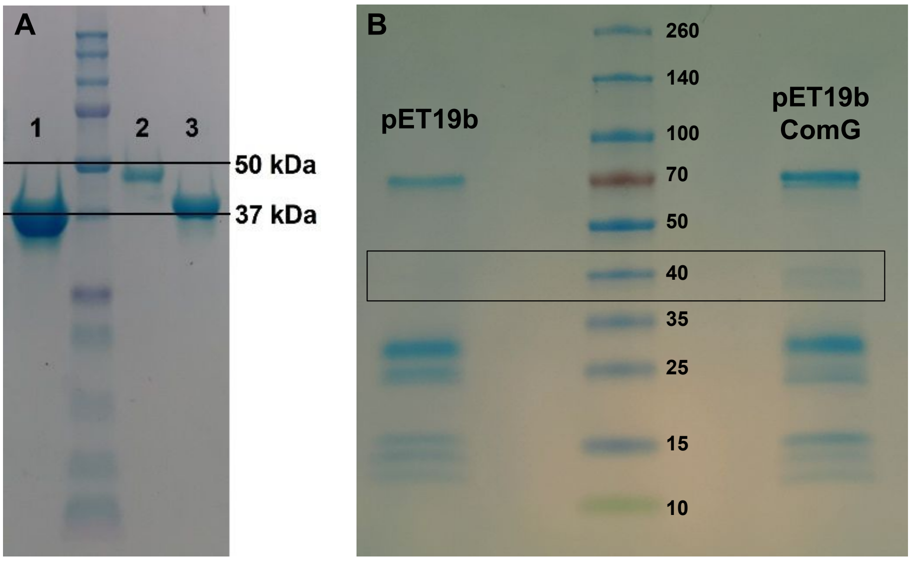

Figure S20. SDS-PAGE of heterologously expressed ComG homologues Nr. 1, 2 and 3 in pET28b+ (numbering according to Table S7, **A**) and ComG in pET19b (**B**).

Supplementary Tables

Table S1. Inhibitory concentrations of corramycin and different reference antibiotics against wild-type *Escherichia coli coli* BL21 (DE3) and *E. coli* expressing ComG. Wild-type *E. coli* was previously transformed with the empty expression vector (pET19b).

**Antibiotic**	**Inhibitory concentration [µg mL^{-1}]**	
	***E. coli* wild-type**	***E. coli* ComG**
Corramycin	1	256
Gentamicin	2	2
Kanamycin	4	4
Tobramycin	2	2
Amikacin	4	4
Neomycin	4	4
Clarithromycin	64	64
Erythromycin	64	64
Ciprofloxacin	0.008	0.008
Levofloxacin	0.0156	0.0156
Capreomycin	64	64

Table S2. Mass list of Cor-P fragmentation in tandem MS/MS analysis.

**b9**	**1160.50**	**y9**	**1161.50**
**b8**	1047.42	y8	994.43
**b7**	932.35	y7	753.39
**b6**	845.32	y6	592.31
**b5**	730.26	y5	535.29
**b4**	673.24	y4	420.22
**b3**	512.15	y3	333.19
**b2**	271.12	y2	218.13
**b1**	104.05	y1	105.04

Table S3. Mass list of corramycin fragmentation in tandem MS/MS analysis.

**b9**	**1081.19**	**y9**	**1082.18**
**b8**	968.04	y8	915.01
**b7**	852.90	y7	753.39
**b6**	765.82	y6	592.31
**b5**	650.69	y5	535.29
**b4**	593.63	y4	420.22
**b3**	432.43	y3	333.19
**b2**	271.27	y2	218.13
**b1**	104.05	y1	105.04

Table S4. Data collection and refinement statistics.

	**ComG apo**	**ComG^C**
**PDB code**	8AHD	8AGY
**Data collection**		
Space group	$P2_1$	$P2_1$
Cell dimensions		
a, *b*, *c* (Å)	50.1, 69.9, 117.6	43.0, 67.1, 75.6
a, b, g (°)	90.0, 98.1, 90.0	90.0, 93.3, 90.0
Wavelength (Å)	0.9724	0.9780
Resolution (Å)	116.4 – 2.10 (2.21 – 2.10)	42.89– 1.50 (1.58 – 1.50)
R_{merge}	0.10 (0.45)	0.06 (0.44)
$CC_{1/2}$	1.00 (0.89)	1.00 (0.96)
$I / \sigma I$	7.9 (2.3)	22.7 (5.5)
Completeness (%)	97.1 (97.9)	98.8 (98.5)
Redundancy	4.5 (4.6)	6.8 (6.6)
**Refinement**		
Resolution (Å)	59.92 – 2.10	38.3 – 1.5
No. reflections	45544 (4558)	67756 (6673)
R_{work} / R_{free}	0.208 / 0.267	0.175 / 0.194
No. atoms	5811	3272
Protein	5383	2775
Ligand/ion	28	95
Water	416	402
B-factors	32.05	24.81
Protein	31.87	23.19
Ligand/ion	30.23	36.33
Water	34.30	33.27
R.m.s. deviations		
Bond lengths (Å)	0.011	0.028
Bond angles (°)	1.21	1.42

Statistics for the highest resolution shell are shown in parentheses.

Table S5. Primers used for cloning of *comG*.

**gene name**	**direction**	**primer**
comG	forward	CTTCATATGGAAAACCTGTATTTTCAGGGCGGCATGGGTAAC AACTCGCGT
	reverse	CTTAAGCTTTTAAGGACCCGGCGGCTT

Table S6. Structurally resolved APHs containing an adenine or guanine base as cofactor (PDB IDs are given) from the Pfam family PF01636 (phosphotransferase enzyme family).

	**PDB IDs**	**Total**
**Adenine base**	1J7L, 1J7U, 1L8T, 2B0Q, 2BKK, 2OLC, 2PUI, 2PUL, 2PUN, 2PUP, 2PYW, 2Q83, 3ATT, 3HAV, 3I0O, 3I0Q, 3TM0, 3W0N, 3W0O, 3W0P, 3W0Q, 3W0R, 3W0S, 4DCA, 4DT8, 4DTA, 4EJ7, 4N57, 4OCK, 4OCP, 4OCV, 4WH2, 4WH3, 6FUX, 6SUL, 6SUM, 6SUN, 6SV5, 7F0B, 7S3L	40
**Guanine base**	3TDV, 3TDW, 4DT9, 4DTB, 4ORK, 5BYL, 5IGI, 5IGJ, 5IGP, 5IGR, 5IGS, 5IGT, 5IGV, 5IGW, 5IGY, 5IGZ, 5IH0, 5IH1, 5IQA, 5IQB, 5IQC, 5IQD, 5IQE, 5IQF, 5IQG, 5IQH, 5IQI, 5UXC, 6C5U, 6CAV, 6CEY, 6CGD, 6CGG, 6CH4, 6CTZ, 7W15, 7W1A	37

Table S7. Significant hits from ESKAPE pathogens. Inhibitory concentration of corramycin against wild-type *Escherichia coli coli* BL21 (DE3) and *E. coli* expressing ComG and ComG homologues. Wild-type *E. coli* was previously transformed with the empty expression vector (pET28b+).

ComG homologue	NCBI identifier	Source ComG/ homologue	Sequence identity [%]	Coverage [%]	E-value	Inhibitory concentration (µg mL^{-1})
-	*E. coli* BL21 (DE3)	-	-	-	-	1
-	*E. coli* ComG	*Corallococcus coralloides* ST201330	-	-	-	> 64
1	RKHS01000004	*Enterobacter* sp. BIGb0359	22.5	59.3	0.004	1
2	RXKH01000023	*Acinetobacter* sp. isolate AWTP1-36	22.6	91.3	$3.61 \cdot 10^{-12}$	1
3	JAHSTX010000 002	*Pseudomonas triticicola* strain SWRI88 2	26.1	91.0	$8.79 \cdot 10^{-7}$	1

Table S8. Overview of the AME-encoding gene profiles and resistance phenotypes of *Escherichia coli* clinical isolates used in this study. AMK: amikacin; GEN: gentamicin; KAN: kanamycin; NEO: neomycin; TOB: tobramycin; no.: number; ^{R}: resistance.

AME-encoding gene profiles	resistance phenotypes	no. of isolates
APH(3')-Ia	KAN^{R}, NEO^{R}	9
AAC(3)-II	GEN^{R}, TOB^{R}	15
AAC(6')-IL	AMK^{R}, KAN^{R}, TOB^{R}	1
AAC(6')-Ib		1
AAC(6')-Ib-cr		11
AAC(3)-II / AAC(6')-Ib(-cr)	AMK^{R}, GEN^{R}, KAN^{R}, TOB^{R}	17
AAC(3)-IIa / APH(3')-Ia	GEN^{R}, KAN^{R}, TOB^{R}	1

Table S9. Antimicrobial resistance profile of the *Escherichia coli* clinical isolate showing resistance to corramycin (resistance genes were identified using ResFinder[37]).

phenotype	resistance marker	
	**gene**	**protein**
sulfonamide resistance	*sul2*	sulfonamide-resistant dihydropteroate synthase
phenicol resistance	*catA1*	chloramphenicol acetyltransferase
disinfectant resistance	*sitABCD*	manganese and iron transporter
aminoglycoside resistance	*aadA5*	aminoglycoside nucleotidyltransferase
	aph(6)-Id	aminoglycoside phosphotransferase
	aph(3')-Ia	
	aph(3'')-Ib	
quinolone resistance	*qnrS1*	quinolone resistance protein (protection of gyrase and topoisomerase IV)
trimethoprim resistance	*dfrA17*	dihydrofolate reductase
tetracycline resistance	*tetB*	tetracycline efflux pump
beta-lactam resistance	*blaTEM-1B*	narrow-spectrum beta-lactamase

References

(1) Sambrook, J.; Russell, D. W. *Molecular Cloning: A Laboratory Manual*, 3rd ed.; Cold Spring Harbor Laboratory Press: Cold Spring Harbor (N.Y.), 2001.

(2) Mancini, S.; Marchesi, M.; Imkamp, F.; Wagner, K.; Keller, P. M.; Quiblier, C.; Bodendoerfer, E.; Courvalin, P.; Böttger, E. C. Population-Based Inference of Aminoglycoside Resistance Mechanisms in Escherichia Coli. *EBioMedicine* **2019**, *46*, 184–192. https://doi.org/10.1016/j.ebiom.2019.07.020.

(3) Patel, J. B.; Clinical and Laboratory Standards Institute. *Performance Standards for Antimicrobial Susceptibility Testing*; 2017.

(4) Gasteiger, E.; Hoogland, C.; Gattiker, A.; Duvaud, S.; Wilkins, M. R.; Appel, R. D.; Bairoch, A. Protein Identification and Analysis Tools on the ExPASy Server. In *The Proteomics Protocols Handbook*; Walker, J. M., Ed.; Humana Press: Totowa, NJ, 2005; pp 571–607. https://doi.org/10.1385/1-59259-890-0:571.

(5) Kabsch, W. Integration, Scaling, Space-Group Assignment and Post-Refinement. *Acta Crystallogr D Biol Crystallogr* **2010**, *66* (2), 133–144. https://doi.org/10.1107/S0907444909047374.

(6) Evans, P. Scaling and Assessment of Data Quality. *Acta Crystallogr D Biol Crystallogr* **2006**, *62* (1), 72–82. https://doi.org/10.1107/S0907444905036693.

(7) Evans, P. R.; Murshudov, G. N. How Good Are My Data and What Is the Resolution? *Acta Crystallogr D Biol Crystallogr* **2013**, *69* (7), 1204–1214. https://doi.org/10.1107/S0907444913000061.

(8) Collaborative Computational Project, Number 4. The CCP4 Suite: Programs for Protein Crystallography. *Acta Crystallogr D Biol Crystallogr* **1994**, *50* (5), 760–763. https://doi.org/10.1107/S0907444994003112.

(9) Terwilliger, T. C.; Adams, P. D.; Read, R. J.; McCoy, A. J.; Moriarty, N. W.; Grosse-Kunstleve, R. W.; Afonine, P. V.; Zwart, P. H.; Hung, L.-W. Decision-Making in Structure Solution Using Bayesian Estimates of Map Quality: The *PHENIX AutoSol* Wizard. *Acta Crystallogr D Biol Crystallogr* **2009**, *65* (6), 582–601. https://doi.org/10.1107/S0907444909012098.

(10) Emsley, P.; Lohkamp, B.; Scott, W. G.; Cowtan, K. Features and Development of *Coot*. *Acta Crystallogr D Biol Crystallogr* **2010**, *66* (4), 486–501. https://doi.org/10.1107/S0907444910007493.

(11) Afonine, P. V.; Grosse-Kunstleve, R. W.; Echols, N.; Headd, J. J.; Moriarty, N. W.; Mustyakimov, M.; Terwilliger, T. C.; Urzhumtsev, A.; Zwart, P. H.; Adams, P. D. Towards Automated Crystallographic Structure Refinement with *Phenix.Refine*. *Acta Crystallogr D Biol Crystallogr* **2012**, *68* (4), 352–367. https://doi.org/10.1107/S0907444912001308.

(12) McCoy, A. J.; Grosse-Kunstleve, R. W.; Adams, P. D.; Winn, M. D.; Storoni, L. C.; Read, R. J. *Phaser* Crystallographic Software. *J Appl Crystallogr* **2007**, *40* (4), 658–674. https://doi.org/10.1107/S0021889807021206.

(13) Chen, V. B.; Arendall, W. B.; Headd, J. J.; Keedy, D. A.; Immormino, R. M.; Kapral, G. J.; Murray, L. W.; Richardson, J. S.; Richardson, D. C. *MolProbity*: All-Atom Structure Validation for Macromolecular Crystallography. *Acta Crystallogr D Biol Crystallogr* **2010**, *66* (1), 12–21. https://doi.org/10.1107/S0907444909042073.

(14) Krissinel, E.; Henrick, K. Inference of Macromolecular Assemblies from Crystalline State. *Journal of Molecular Biology* **2007**, *372* (3), 774–797. https://doi.org/10.1016/j.jmb.2007.05.022.

(15) Holm, L. Dali Server: Structural Unification of Protein Families. *Nucleic Acids Research* **2022**, *50* (W1), W210–W215. https://doi.org/10.1093/nar/gkac387.

(16) Jo, S.; Kim, T.; Iyer, V. G.; Im, W. CHARMM-GUI: A Web-Based Graphical User Interface for CHARMM. *J. Comput. Chem.* **2008**, *29* (11), 1859–1865. https://doi.org/10.1002/jcc.20945.

(17) Lee, J.; Cheng, X.; Swails, J. M.; Yeom, M. S.; Eastman, P. K.; Lemkul, J. A.; Wei, S.; Buckner, J.; Jeong, J. C.; Qi, Y.; Jo, S.; Pande, V. S.; Case, D. A.; Brooks, C. L.; MacKerell, A. D.; Klauda, J. B.; Im, W. CHARMM-GUI Input Generator for NAMD, GROMACS, AMBER, OpenMM, and CHARMM/OpenMM Simulations Using the CHARMM36 Additive Force Field. *J. Chem. Theory Comput.* **2016**, *12* (1), 405–413. https://doi.org/10.1021/acs.jctc.5b00935.

(18) Huang, J.; Rauscher, S.; Nawrocki, G.; Ran, T.; Feig, M.; de Groot, B. L.; Grubmüller, H.; MacKerell, A. D. CHARMM36m: An Improved Force Field for Folded and Intrinsically Disordered Proteins. *Nat Methods* **2017**, *14* (1), 71–73. https://doi.org/10.1038/nmeth.4067.

(19) Vanommeslaeghe, K.; Hatcher, E.; Acharya, C.; Kundu, S.; Zhong, S.; Shim, J.; Darian, E.; Guvench, O.; Lopes, P.; Vorobyov, I.; Mackerell, A. D. CHARMM General Force Field: A Force Field for Drug-like Molecules Compatible with the CHARMM All-Atom Additive Biological Force Fields. *J. Comput. Chem.* **2009**, NA-NA. https://doi.org/10.1002/jcc.21367.

(20) Jorgensen, W. L.; Chandrasekhar, J.; Madura, J. D.; Impey, R. W.; Klein, M. L. Comparison of Simple Potential Functions for Simulating Liquid Water. *The Journal of Chemical Physics* **1983**, *79* (2), 926–935. https://doi.org/10.1063/1.445869.

(21) Van Der Spoel, D.; Lindahl, E.; Hess, B.; Groenhof, G.; Mark, A. E.; Berendsen, H. J. C. GROMACS: Fast, Flexible, and Free. *J. Comput. Chem.* **2005**, *26* (16), 1701–1718. https://doi.org/10.1002/jcc.20291.

(22) Humphrey, W.; Dalke, A.; Schulten, K. VMD: Visual Molecular Dynamics. *Journal of Molecular Graphics* **1996**, *14* (1), 33–38. https://doi.org/10.1016/0263-7855(96)00018-5.

(23) Nosé, S. A Molecular Dynamics Method for Simulations in the Canonical Ensemble. *Molecular Physics* **1984**, *52* (2), 255–268. https://doi.org/10.1080/00268978400101201.

(24) Hoover, W. G. Canonical Dynamics: Equilibrium Phase-Space Distributions. *Phys. Rev. A* **1985**, *31* (3), 1695–1697. https://doi.org/10.1103/PhysRevA.31.1695.

(25) Parrinello, M.; Rahman, A. Polymorphic Transitions in Single Crystals: A New Molecular Dynamics Method. *Journal of Applied Physics* **1981**, *52* (12), 7182–7190. https://doi.org/10.1063/1.328693.

(26) Darden, T.; York, D.; Pedersen, L. Particle Mesh Ewald: An $N \cdot \log(N)$ Method for Ewald Sums in Large Systems. *The Journal of Chemical Physics* **1993**, *98* (12), 10089–10092. https://doi.org/10.1063/1.464397.
(27) Essmann, U.; Perera, L.; Berkowitz, M. L.; Darden, T.; Lee, H.; Pedersen, L. G. A Smooth Particle Mesh Ewald Method. *The Journal of Chemical Physics* **1995**, *103* (19), 8577–8593. https://doi.org/10.1063/1.470117.
(28) Hess, B.; Bekker, H.; Berendsen, H. J. C.; Fraaije, J. G. E. M. LINCS: A Linear Constraint Solver for Molecular Simulations. *J. Comput. Chem.* **1997**, *18* (12), 1463–1472. https://doi.org/10.1002/(SICI)1096-987X(199709)18:12<1463::AID-JCC4>3.0.CO;2-H.
(29) Miyamoto, S.; Kollman, P. A. Settle: An Analytical Version of the SHAKE and RATTLE Algorithm for Rigid Water Models. *J. Comput. Chem.* **1992**, *13* (8), 952–962. https://doi.org/10.1002/jcc.540130805.
(30) Altschul, S. F.; Gish, W.; Miller, W.; Myers, E. W.; Lipman, D. J. Basic Local Alignment Search Tool. *Journal of Molecular Biology* **1990**, *215* (3), 403–410. https://doi.org/10.1016/S0022-2836(05)80360-2.
(31) Seemann, T. Prokka: Rapid Prokaryotic Genome Annotation. *Bioinformatics* **2014**, *30* (14), 2068–2069. https://doi.org/10.1093/bioinformatics/btu153.
(32) Katoh, K.; Standley, D. M. MAFFT Multiple Sequence Alignment Software Version 7: Improvements in Performance and Usability. *Molecular Biology and Evolution* **2013**, *30* (4), 772–780. https://doi.org/10.1093/molbev/mst010.
(33) Price, M. N.; Dehal, P. S.; Arkin, A. P. FastTree 2 – Approximately Maximum-Likelihood Trees for Large Alignments. *PLoS ONE* **2010**, *5* (3), e9490. https://doi.org/10.1371/journal.pone.0009490.
(34) Crooks, G. E.; Hon, G.; Chandonia, J.-M.; Brenner, S. E. WebLogo: A Sequence Logo Generator: Figure 1. *Genome Res.* **2004**, *14* (6), 1188–1190. https://doi.org/10.1101/gr.849004.
(35) Sievers, F.; Higgins, D. G. Clustal Omega for Making Accurate Alignments of Many Protein Sequences. *Protein Sci* **2018**, *27* (1), 135–145. https://doi.org/10.1002/pro.3290.
(36) Blin, K.; Shaw, S.; Kloosterman, A. M.; Charlop-Powers, Z.; van Wezel, G. P.; Medema, M. H.; Weber, T. AntiSMASH 6.0: Improving Cluster Detection and Comparison Capabilities. *Nucleic Acids Research* **2021**, *49* (W1), W29–W35. https://doi.org/10.1093/nar/gkab335.
(37) Florensa, A. F.; Kaas, R. S.; Clausen, P. T. L. C.; Aytan-Aktug, D.; Aarestrup, F. M. ResFinder – an Open Online Resource for Identification of Antimicrobial Resistance Genes in next-Generation Sequencing Data and Prediction of Phenotypes from Genotypes. *Microbial Genomics* **2022**, *8* (1). https://doi.org/10.1099/mgen.0.000748.
